# Hyperoxia/Hypoxia Exposure Primes a Sustained Pro-Inflammatory Profile of Preterm Infant Macrophages Upon LPS Stimulation

**DOI:** 10.3389/fimmu.2021.762789

**Published:** 2021-11-18

**Authors:** Nele Twisselmann, Julia Pagel, Axel Künstner, Markus Weckmann, Annika Hartz, Kirsten Glaser, Anne Hilgendorff, Wolfgang Göpel, Hauke Busch, Egbert Herting, Jason B. Weinberg, Christoph Härtel

**Affiliations:** ^1^ Department of Pediatrics, University of Lübeck and University Medical Center Schleswig-Holstein, Lübeck, Germany; ^2^ Department of Infectious Diseases and Microbiology, University of Lübeck and University Medical Center Schleswig-Holstein, Lübeck, Germany; ^3^ Medical Systems Biology Group, Institute of Experimental Dermatology, University of Lübeck and University Medical Center Schleswig-Holstein, Lübeck, Germany; ^4^ Department of Pediatrics Pneumology & Allergology, University Medical Center Schleswig-Holstein, Lübeck, Germany; ^5^ Airway Research Center North (ARCN) , Member of the German Center for Lung Research (DZL), Lübeck, Germany; ^6^ Center for Pediatric Research, Division of Neonatology, Department of Women’s and Children’s Health, University of Leipzig Medical Centre, Leipzig, Germany; ^7^ Center for Comprehensive Developmental Care (CDeCLMU), Member of the German Center for Lung Research (DZL), Hospital of the Ludwig-Maximilians University (LMU), CPC-M bioArchive, Munich, Germany; ^8^ Department of Pediatrics, University of Michigan, Ann Arbor, MI, United States; ^9^ Department of Microbiology and Immunology, University of Michigan, Ann Arbor, MI, United States; ^10^ Department of Pediatrics, University of Würzburg, Würzburg, Germany

**Keywords:** preterm infants, sustained inflammation, macrophages, hyperoxia, hypoxia, infection, bronchopulmonary dysplasia

## Abstract

Preterm infants are highly susceptible to sustained lung inflammation, which may be triggered by exposure to multiple environmental cues such as supplemental oxygen (O_2_) and infections. We hypothesized that dysregulated macrophage (MФ) activation is a key feature leading to inflammation-mediated development of bronchopulmonary dysplasia (BPD) in preterm infants. Therefore, we aimed to determine age-dependent differences in immune responses of monocyte-derived MФ comparing cord blood samples derived from preterm (n=14) and term (n=19) infants as well as peripheral blood samples from healthy adults (n=17) after lipopolysaccharide (LPS) exposure. Compared to term and adult MФ, LPS-stimulated preterm MФ showed an enhanced and sustained pro-inflammatory immune response determined by transcriptome analysis, cytokine release inducing a RORC upregulation due to T cell polarization of neonatal T cells, and TLR4 surface expression. In addition, a double-hit model was developed to study pulmonary relevant exposure factors by priming MФ with hyperoxia (O_2_ = 65%) or hypoxia (O_2_ = 3%) followed by lipopolysaccharide (LPS, 100ng/ml). When primed by 65% O_2_, subsequent LPS stimulation in preterm MФ led to an exaggerated pro-inflammatory response (e.g. increased HLA-DR expression and cytokine release) compared to LPS stimulation alone. Both, exposure to 65% or 3% O_2_ together with subsequent LPS stimulation, resulted in an exaggerated pro-inflammatory response of preterm MФ determined by transcriptome analysis. Downregulation of two major transcriptional factors, early growth response gene (Egr)-2 and growth factor independence 1 (Gfi1), were identified to play a role in the exaggerated pro-inflammatory response of preterm MФ to LPS insult after priming with 65% or 3% O_2_. Preterm MФ responses to LPS and hyperoxia/hypoxia suggest their involvement in excessive inflammation due to age-dependent differences, potentially mediated by downregulation of Egr2 and Gfi1 in the developing lung.

## Introduction

Preterm infants are faced with the challenge of encountering the *ex-utero* environment with a premature immune system and a premature lung (1–4). Environmental cues, such as infection and supplemental oxygen (O_2_), can trigger a sustained pro-inflammatory dysregulation of the premature lung immune system leading to an arrest of lung development over time with reduced alveolarization and vascularization, which is termed bronchopulmonary dysplasia (BPD) (5–10). BPD is a major complication of premature birth and has been linked to chronic pulmonary inflammation with a high risk of mortality and long-term morbidities (10–16). An improved understanding of the mechanisms causing BPD development is needed to facilitate the development of new preventive and therapeutic strategies.

Recent animal models have investigated lung immunity and development after birth using a sequential double-hit model that mimics inflammation and hyperoxia (17–20). In those models, the pro-inflammatory immune response and the disruption of lung development were exaggerated after double-hit exposure. For the infant lung, macrophages (MФ) constitute a crucial immune cell population with a dual role for tissue development and immune defense (4, 21). We hypothesize that MФ are important cellular components that mediate the development of lung disease in preterm infants. This notion is supported by a study from Blackwell et al describing reduced branching in embryonic lung explants due to NFkB-activated MФ (22).

Few studies have investigated the immunological response and function of neonatal human MФ. Previous studies have characterized various stimulus-response behaviors of human monocyte-derived MФ from term infants and demonstrated differences in immune responses compared to responses of adult MФ *in vitro.* Wisgrill et al showed a similar immune response of term MФ compared to adult MФ following stimulation with lipopolysaccharide (LPS) or lipoteichoic acid (23). Another study demonstrated enhanced Fc receptor-dependent phagocytosis in term MФ compared to adult MФ (24). After polarization with interferon gamma (IFNγ) and interleukin (IL-)10, term MФ showed an impaired phenotype and function as well as a diminished glycolytic metabolism compared to adult MФ (25, 26). Other investigations found a correlation of immune function in preterm monocytes with gestational age. The immune function increased with gestational age towards term infant levels and was influenced by exposure to infection (27–31). Yet, there is a paucity of data regarding neonatal MФ function in the context of prematurity, largely due to challenges with sample collection and limited amounts of available biomaterial from infants.

Here, we developed a sequential double-hit model by priming monocyte-derived MФ from preterm infants with varying oxygen concentrations to simulate hyperoxia and hypoxia and using sequential LPS exposure to stimulate infection. This double-hit model mimics key lung exposure factors previously shown to contribute to an increased risk for BPD development in preterm infants. The primary objective of our explorative study was to investigate developmental differences in the immune responses of MФ. We hypothesized that human MФ show a gestational age-dependent difference in their immune response, which can lead to detrimental pro-inflammatory responses in preterm infants.

## Materials and Methods

### Study Cohort

We performed an explorative study in the Department of Pediatrics at the University Hospital of Lübeck, a tertiary level perinatal center for the treatment of high-risk neonates, as part of our Immunoregulation of the Newborn (IRoN) study. Cord blood samples and clinical data were obtained from infants born between May 1^st^, 2017 and January 31^st^, 2019. The inclusion criteria were preterm infants with gestational age between 30 + 0 and 34 + 6 weeks without lethal abnormalities. Cord blood samples from late preterm and term infants born with a gestational age above 36 + 0 weeks and peripheral blood samples from healthy adult donors served as controls.

### Study Approval

Written informed consent was obtained from parents or legal guardians on behalf of the infants as well as adult donors (> 18 years) enrolled into our study. The study was approved by the local committee on research in human subjects at the University of Lübeck (IRON AZ 15-304). All blood samples were obtained according to current guidelines of the European Medical Agency on the investigation of medicinal products in term and preterm infants; Committee for Medicinal Products for Human Use and Pediatric Committee (PDCO, 2009).

### Definitions

Gestational age was calculated from the best obstetric estimate based on early prenatal ultrasound and obstetric examination.

Late-onset sepsis (LOS) was defined by clinical presentation or results of blood culture, occurring after the first 72 h of life. Clinical LOS was defined as a condition prompting a neonatologist to treat an infant with antibiotics that were continued for at least 5 days.

Intra-amniotic infection syndrome (AIS) was defined as labour ± rupture of membranes, increased maternal inflammatory markers without any other cause (C-reactive protein > 10 mg/l or elevation of white blood cell count > 16000/µl), maternal fever (≥ 38.0°C), fetal or maternal tachycardia, painful uterus and foul-smelling amniotic liquor.

### Sample Collection

All blood samples analyzed in this study were collected using EDTA as an anticoagulant (S monovettes K3E). Cord blood was collected by the attending physician or midwife immediately after the infant was born. Adult blood samples were collected by peripheral vein puncture. All samples were processed within 24 h.

### Cell Isolation

Monocytes were isolated directly from whole blood by immunomagnetic negative selection using the EasySep™ Direct Human Monocyte Isolation Kit (Stemcell Technologies, Vancouver, Canada) according to the manufacturer’s instructions.

CD4+ T cells were isolated directly from cord blood of term infants by immunomagnetic negative selection using the EasySep™ Direct Human CD4+ T Cell Isolation Kit (Stemcell Technologies, Vancouver, Canada). Subsequent CD25 depletion was performed using the EasySep™ Human CD25 Positive Selection Kit (Stemcell Technologies, Vancouver, Canada) according to the manufacturer’s instructions.

### Differentiation Into Monocyte-Derived Macrophages

Following monocyte isolation, 5x10^5^ to 7.5x10^5^ cells per cm^2^ were seeded and differentiation medium was added to achieve a total volume of 1 mL medium per cm^2^. The differentiation medium contained RPMI-1640 medium (without glutamine; Gibco^®^, Thermo Fisher Scientific, Waltham, MA, USA) with 10% fetal bovine serum (FBS; PAN-Biotech, Aidenbach, Germany), 1M HEPES buffer solution (PAN-Biotech, Aidenbach, Germany), 10 mg/mL L-glutamine (Lonza, Veriers, Belgium), 1x Penicillin (10000U)/Streptomycin (10mg/ml) (P/S; Sigma-Aldrich Corporation, St. Louis, USA), 25mM β-mercaptoethanol (β-ME; AppliChem GmbH, Darmstadt, Germany). Cell counting was performed in a Neubauer counting chamber (depth 0.1 mm) using trypan blue (0.4%) to test viability (always > 95%). After a 1 to 2 h resting and adherence phase in an incubator containing an atmosphere of 5% CO_2_ and 21% O_2_ at a temperature of 37°C, the medium was replaced with differentiation medium additionally containing 10 ng/mL recombinant human macrophage colony-stimulating factor (M-CSF; PeproTech, Rocky Hill, NJ, USA), and cells were placed back in the incubator. Following 3 days of differentiation, the medium was replaced with fresh medium containing 10 ng/mL of M-CSF, and cells were incubated for another 3 days.

### Double-Hit Model

**Figure 1:**
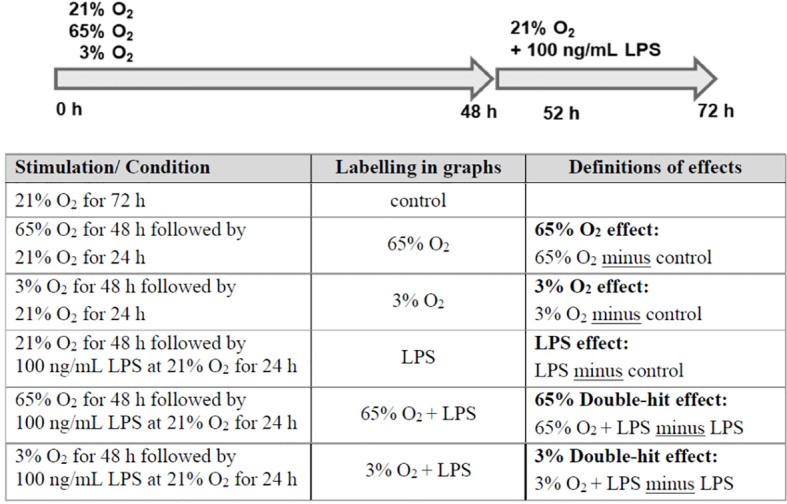
Sequential double-hit model for lung inflammation. Monocyte-derived macrophages were incubated for 48 h in different O_2_ concentrations (21% atmospheric O_2_, 3% O_2_, or 65% O_2_). Subsequently, cells were removed from the different O_2_ conditions and stimulated with 100 ng/mL LPS for 4 h (52 h time point) or 24 h (72 h time point) in atmospheric O_2_.

To detach MФ after differentiation, Accutase (Gibco^®^, Thermo Fisher Scientific, Waltham, MA, USA) was used according to the manufacturer’s instructions. 4.5 to 5.0x10^5^ cells/mL were seeded in 200 µL stimulation medium per well in a flat-bottom 96-well plate for RNA isolation or in 400 µL per FACS tube for flow cytometry. The stimulation medium contained RPMI-1640 medium (without glutamine; Gibco^®^, Thermo Fisher Scientific, Waltham, MA, USA) with 10% fetal bovine serum (FBS; PAN-Biotech, Aidenbach, Germany), and 10 mg/mL L-glutamine (Lonza, Veriers, Belgium). After a 1 to 2 h resting and adherence phase in an incubator containing an atmosphere of 5% CO_2_ and 21% O_2_ at 37°C, cells were placed in different incubators with 5% CO_2_ at 37°C containing 21% atmospheric O_2_, 3% O_2_ (incubator with N_2_-regulation), or 65% O_2_ (air-tight sealed chamber filled with gas mixture) and incubated for 48 h. Subsequently, cells were removed from the different O_2_ conditions and stimulated with 100 ng/mL LPS for 4 h (52 h time point) or 24 h (72 h time point) in atmospheric O_2_ with 5% CO_2_ at 37°C. Unstimulated cells served as controls. This sequential double-hit stimulation model is depicted in **Figure 1**. After the LPS stimulation, supernatant was harvested on ice and stored at -80°C until further use. Cells were used for RNA isolation or flow cytometry.

### Co-Culture of T Cells With Supernatants From Stimulated Macrophages

Following CD4+ CD25- isolation and a 1 h resting phase at 37°C in differentiation medium supplemented with 1500 U/mL IL-2 (to achieve an end concentration of 100 U/mL IL-2), 2x10^4^ cells were seeded in 10 µL per well in a round-bottom 96-well plate. For culturing T cells, Treg inspector beads loaded with biotinylated CD3/CD28/CD2 antibodies (Miltenyi Biotec, Bergisch Gladbach, Germany) were used according to the manufacturer’s instructions (1 bead per cell). 140 µL of differentiation medium was added to negative controls (without beads) and medium controls (with beads), which were performed in triplicate for normalization. 140 µL of supernatant from experiments using preterm, term, or adult MФ (described above) was added to all other wells. After 6 days of incubation in atmospheric O_2_ with 5% CO_2_ at 37°C, supernatant from T cells was carefully collected on ice and stored at -80°C, and cells were used for RNA isolation.

### Flow Cytometry

Cells were washed once with 1 mL PBS buffer. Afterwards, 20 µL FcR blocking reagent (1:25, Miltenyi Biotec, Bergisch Gladbach, Germany) was added to cells in 100 µl PBS, samples were pulse vortexed, and then incubated for 15 min at room temperature in the dark. The eFluor^®^780 conjugated Fixable Viability Dye (eBioscience, Thermo Fisher Scientific, Waltham, MA, USA) was pre-diluted 1:100 in PBS buffer. Anti-human antibodies against surface proteins were added: 1:25 PE conjugated CD200R (eBioscience, Thermo Fisher Scientific, Waltham, MA, USA), 1:25 APC conjugated CD206 (eBioscience, Thermo Fisher Scientific, Waltham, MA, USA), 1:25 FITC conjugated CD80 (eBioscience, Thermo Fisher Scientific, Waltham, MA, USA), 1:25 BV421 conjugated CD284 (TLR4, Biolegend, San Diego, CA, USA), 1:25 PerCP/Cy5.5 conjugated HLA-DR (Biolegend, San Diego, CA, USA), and 1:20 pre-diluted Fixable Viability Dye. All samples were pulse vortexed and incubated for 25 min at room temperature in the dark. Subsequently, samples were washed with 2 mL FACS buffer (0.5% BSA, 2mM EDTA in PBS buffer) and resuspended in 150 µL FACS buffer for flow cytometric analysis with the BD FACSCanto™ II using BD FACS Diva™ software. A total of 20,000 events per sample were measured. Colors were compensated using unstained and single-stained controls. To generate a positive heat-killed control, 50µL cell suspension obtained after the first washing step were incubated at 65°C for 2 min, then 2 min on ice and transferred back to half of the remaining living cells. A compensation calculation was performed using BD FACSDiva™ software. Single cells were then determined by gating for FSC-Height (FSC-H) by FSC-Area (FSC-A) *via* exclusion of duplets. Fixable Viability Dye low events were identified as living cells. To further characterize protein expression in MФ, living singlet cells were analyzed for the different marker expression. Mean fluorescence intensity [MFI] was assessed in histograms to determine the shift in fluorescence intensity for the marker expression in the cell population (stained MFI - unstained MFI). Fluorescence minus one (FMO) samples were used as controls.

### Quantitative Analysis of Cytokines in Culture Supernatants

To quantify cytokine release, MФ supernatants were analyzed using the LEGENDplex™ Human M1/M2 Macrophage Panel (10-plex) with V-bottom Kit according to the manufacturer’s instructions (Biolegend, San Diego, CA, USA). The loaded plate was incubated shaking overnight at 4°C. All samples and standards were measured with the BD FACSCanto™ II using BD FACS Diva™ software. A total of 4,000 events of the two bead populations were measured, including approximately 300 events per bead. Gating and analysis were performed using the LEGENDplex v8.0 software according to supplied instructions. The concentration of a particular analyte was determined using a standard curve generated in the same assay.

### RNA Isolation

RNA was isolated using the NucleoSpin RNA Isolation Kit (Macherey-Nagel, Düren, Germany). β-mercaptoethanol was added to the RNA lysis buffer in a 1:100 dilution. Cells of one well in a 96-well plate were lysed with 200-300 µl RNA lysis buffer and stored at -80°C until further use. Isolation was performed according to the manufacturer’s protocol. RNA was eluted using 40 µL RNase-free water and stored at -80°C until further use.

### Reverse Transcription PCR

Complementary DNA (cDNA) was generated from RNA templates. The reaction mix contained 4 µL 5x reaction buffer, 1 µL maxima H minus reverse transcriptase (Thermo Fisher Scientific, Waltham, MA, USA), 2 µL dNTP nucleotide mix (Roche Diagnostics GmbH, Mannheim, Germany), 0.5 µL RiboLock RNase Inhibitor (Thermo Fisher Scientific, Waltham, MA, USA), 5.5 µL nuclease-free water and 5 µL RNA template. PCR reactions were performed using a thermocycler C 1000 (Bio-Rad, Munich, Germany). The PCR temperature profile was: 1) hybridization at 25°C for 10 min, 2) reverse transcription at 50°C for 30 min, 3) enzyme inactivation at 85°C for 5 min, and 4) cooling at 4°C. After cDNA synthesis, the samples were stored at -20°C until further use.

### Quantitative Real-Time PCR

Quantitative real-time PCR was performed with the cDNA in a white 96-well plate using the dye SYBR Green. The amount of cDNA and other required ingredients were used according to LightCycler^®^ 480 SYBR Green I Master Kit instructions (Roche, Penzberg, Germany) as follows: 10 µL master mix, 1 µL primer mix for transcription factor genes (Bio-Rad, Munich, Germany) or 0.2 µl forward and 0.2 µl reverse primer for β-Actin (5´to 3´sequence for human β-Actin: forward CCT GGC ACC CAG CAC AAT and reverse GGG CCG GAC TCG TCA TAC from TIB MOLBIOL, Berlin, Germany), 7.6 or 7.0 µL nuclease-free water and 2 µL cDNA template. Human Transcription factor gene primers for FOXP3 (qHsaCID0007630), GATA3 (qHsaCED0043189), RORC (qHsaCID0008528) and TBX21 (qHsaCED0042343) were used from Bio-Rad, Munich, Germany. The PCR was run in a LightCycler 480 Instrument II (Roche Diagnostics GmbH, Mannheim, Germany) using the following program: 1) initial denaturation at 95°C for 10 min, 2) 45 cycles of denaturation at 95°C for 10 sec, annealing 60°C for 10 sec, and elongation at 72°C for 20 sec, 3) melting curve starting at 95°C for 1 sec going down to 50°C in 30 sec making an acquisition every 5°C, and 4) cooling at 40°C. Evaluation of the data was performed with the LightCycler Data Analysis program. Data were normalized to the corresponding mRNA expression of the housekeeping gene β-Actin. Changes in gene expression were analyzed using the 2^−ΔΔCT^ method.

### RNA Sequencing

Sequencing of total RNA samples isolated from MФ was performed by Novogene (Hong Kong, China). RNA concentration was determined using the Qubit 2.0 fluorometer (Life Technologies), and samples with a total amount of more than 50 ng were used (Qubit RNA HS Assay Kit from Invitrogen GmbH, Darmstadt, Germany). Total RNA sample quality control was performed using Nanodrop as preliminary quantification, agarose gel electrophoresis to test degradation and contamination, and the 2100 Bioanalyzer (Agilent) to check integrity and quantification. After quality control of RNA samples, library preparation was performed using a 250-300 bp insert cDNA library (low-input). Then mRNA was enriched using oligo (dT) beads and randomly fragmented in fragmentation buffer followed by cDNA synthesis using random hexamers and reverse transcriptase. After first-strand synthesis, a custom second-strand synthesis buffer (Illumina) was added with dNTPs, RNase H and *Escherichia coli* polymerase I to generate the second strand by nick-translation. The final cDNA library was ready after a round of purification, terminal repair, A-tailing, ligation of sequencing adapters, size selection and PCR enrichment. Library concentration was quantified using a Qubit 2.0 fluorometer, and then diluted to 1 ng/μL before checking insert size on a 2100 Bioanalyzer and quantifying to greater accuracy by quantitative PCR (library activity > 2nM). Following library preparation, sequencing of the library was performed using an Illumina PE150 platform according to activity and expected data volume. Raw fastq files have been deposited in the European genome-phenome archive (EGA) under the accession number EGAS00001004974.

### Processing and Analysis of Raw RNA Sequencing Data

Quality control of RNA-seq reads was visually inspected using FASTQC. Afterwards, reads were pseudoaligned to human cDNA and ncRNA (Ensembl v94, GRCh38) using KALLISTO (v0.43.1) with 30 bootstrap cycles. On average, 90% of reads (equivalent to 33 million reads) were mapped per sample. Quantification files were imported to the R package and gene expression analyses were performed using DESeq2 (v1.30.0). Within the preterm and term group, pairwise comparisons were performed to determine the LPS effects [LPS stimulated cells to 21% O_2_ (control)], the oxygen effects [65% O_2_ and 3% O_2_ stimulated cells to 21% O_2_ (control)], and the double-hit effects (65% O_2_ + LPS and 3% O_2_ + LPS stimulated cells to LPS). Significantly different expressed genes were selected (p_adj_ < 0.05, unpaired t-test). To analyze the preterm effects in the above mentioned pairwise comparisons, the term effects were subtracted (e.g., preterm LPS effects – term LPS effects). Multi-factorial RNA-seq analysis was performed using DESeq2. Gene set enrichment analysis was performed on log2 fold changes using GAGE (v2.32.0) against REACTOME gene sets (provided by MSIGDB library) for pathway enrichment analysis (p_adj_ < 0.05). In addition, enrichment against the MUELLER PLURINET gene set (32) was tested using GAGE. Principal component analysis (PCA) was performed to inspect the distribution of samples using the mapped RNA-seq data and selecting for the 5,000 most variable genes (R function prcomp with scaling). Pathway enrichment results were plotted using the R package REDER (v1.38.0). To identify gene-pathway regulatory relationships, transcriptional regulatory associations in pathways (TRAP) were analyzed following the approached published by Kwong et al., 2012 (33) using significant gene sets (p_adj_ < 0.1).

### Statistics

Data were analyzed using GraphPad Prism^®^ version 7 except for sequencing data, which were analyzed using R version 3.5.2. After testing for normal distribution in GraphPad Prism^®^, two-way ANOVA followed by Holm-Sidak’s multiple comparisons test was performed comparing conditions within group (e.g., control *versus* LPS within preterm) or comparing groups within condition (e.g., preterm *versus* term within LPS stimulated cells). Matching was done between conditions.

## Results

### Cohort Characteristics

We recruited a cohort of n=14 preterm infants [mean ± SD (range): Gestational age in weeks 33 ± 1 (30.6 – 34.6); birth weight in grams 2016 ± 581 (925 – 3525); female gender n=5 (36%)], n=19 late preterm and term infants [mean ± SD (range): Gestational age in weeks 39 ± 1 (36.1 – 41.4); birth weight in grams 3536 ± 517 (2670 – 4640); female gender n=10 (53%)], and n=17 healthy adults as controls. No inflammatory or other diseases were reported for included patients during the first 72 h of life, except for one intra-amniotic infection in the preterm infant group for cytokine release. All babies were born *via* caesarean section, except for three term infants born vaginally.

### Effects of Gestational Age on MФ Immunological Profile

To determine whether preterm MФ respond differently in an infection model, we first analyzed the effect of LPS exposure on MФ from preterm and term infants as well as adults. We assayed cytokine release, activation marker expression, effects of MФ supernatant on neonatal T helper polarization, and MФ transcriptome analysis. Of note, following differentiation with M-CSF for 6 days, there were no differences in viability (median > 95%), morphology, or protein expression of CD14, CD68 and CD11b between monocyte-derived MФ from preterm infants, term infants, and adults (**Supplementary Methods 1 and 2** and **Supplementary. Figure 1**).

#### Sustained and Enhanced Cytokine Release by Preterm MФ After LPS Stimulation

**Figure 2:**
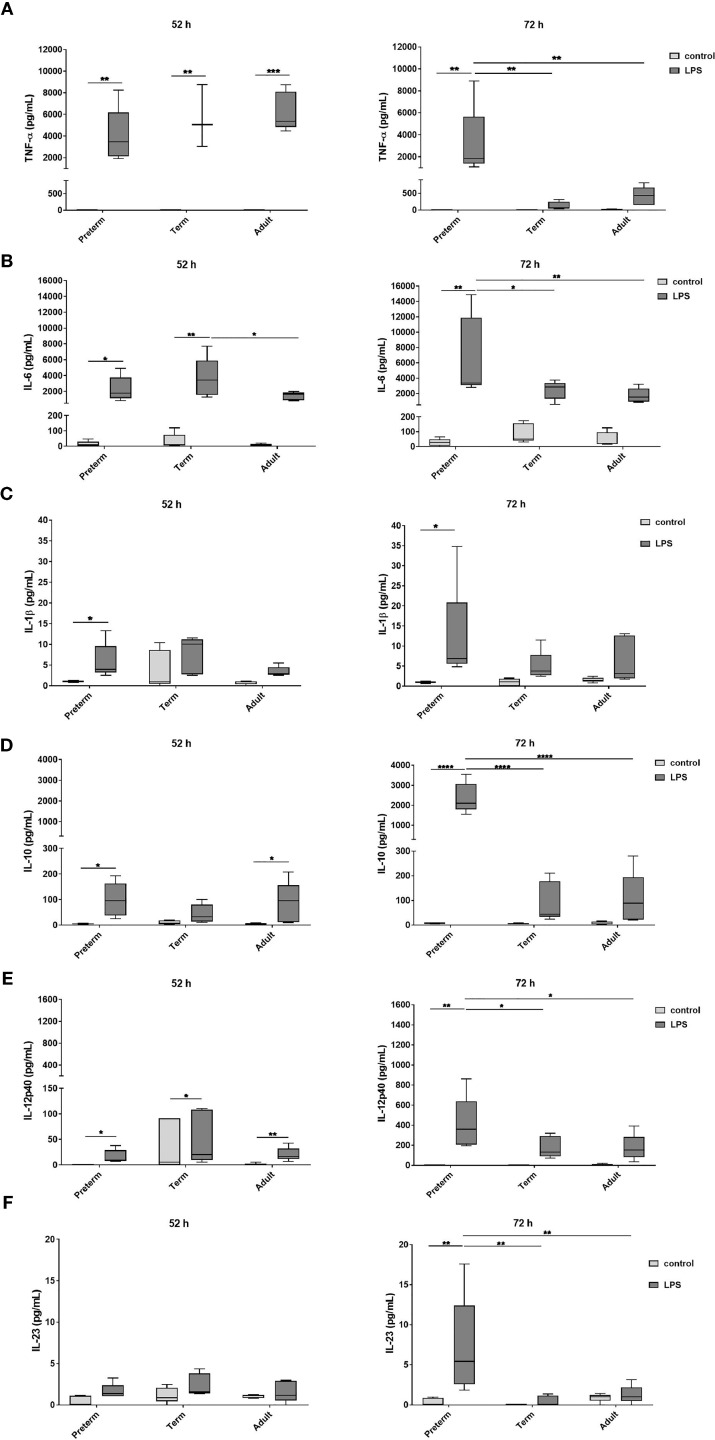
Enhanced cytokine release after 72 h LPS stimulation in preterm macrophages (MФ) compared to term and adult MФ. **(A)** TNFα, **(B)** IL-6, **(C)** IL-1β, **(D)** IL-10, **(E)** IL-12p40, and **(F)** IL-23 release of LPS-stimulated MФ measured in the supernatant after incubation for 52 h and 72 h (48 h 21% O_2_ followed by 4 h or 24 h at 21% O_2_ with or without LPS) using a cytokine bead array (n=5 independent experiments per time point with supernatants of up to ten different donors per group, box plots: median ± 25^th^ to 75^th^ percentiles and minimum to maximum, ANOVA followed by Holm-Sidak’s test, *p<0.05, **p<0.01, ***p<0.001, and ****p<0.0001).

To evaluate developmental differences in cytokine release by MФ after LPS stimulation, we analyzed several representative cytokines (**Figure 2**). At 52 h, MФ production of TNFα, IL-6, and IL-10 was increased to a similar degree upon LPS stimulation in all three age groups. At 72 h, preterm MФ showed a sustained LPS-induced release of TNFα and IL-6 (**Figures 2A, B**). However, term and adult MФ released significantly lower amounts of TNFα and IL-6 after LPS stimulation for 72 h compared to preterm MФ (**Figures 2A, B**). In addition, LPS-induced production of IL-1β, IL-10, IL-12p40 and IL-23 was increased only for preterm MФ (**Figures 2C–F**). IL12p70 and IFNγ release were not detected in any group (data not shown). In summary, LPS-stimulated cytokine release by preterm MФ was sustained and enhanced compared to term and adult MФ.

#### Effects of LPS Stimulation on Expression of MФ Activation Markers

**Figure 3:**
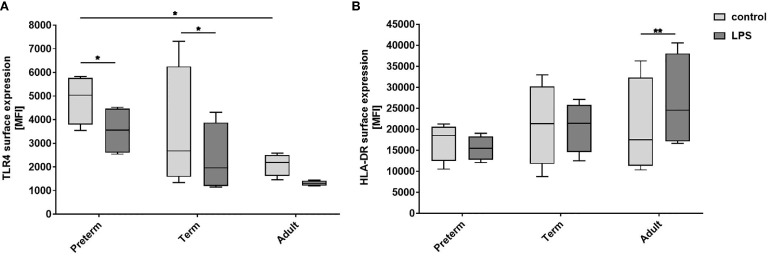
Expression of activation markers on preterm, term, and adult macrophages (MФ) after LPS stimulation. LPS-stimulated MФ of preterm infants compared to term and adult MФ incubated for 72 h (48 h 21% O_2_ followed by 24 h at 21% O_2_ with or without LPS). **(A)** Surface expression of TLR4. **(B)** Surface expression of HLA-DR. Mean fluorescence intensity [MFI] of surface proteins on macrophages in all three groups assessed by flow cytometry. (n=4 independent experiments with cells of four different donors, box plots: median ± 25^th^ to 75^th^ percentiles and minimum to maximum, ANOVA followed by Holm-Sidak’s test, *p<0.05, and **p<0.01).

Next, we analyzed surface expression of cluster of differentiation (CD)80, CD200R, CD206, Toll-like receptor (TLR)4, and human leukocyte antigen - DR isotype (HLA-DR) to determine whether there are developmental differences in LPS-induced MФ responses. The first three markers have been used by Jaguin et al to characterize the pro- and anti-inflammatory state of human MDMs (34). Jaguin et al characterized pro-inflammatory LPS/IFNγ-stimulated human MDMs by upregulation of costimulatory T cell activation marker CD80 (pro-inflammatory marker), downregulation of regulatory CD200R (anti-inflammatory marker) and no expression difference of mannose receptor CD206 (inflammatory marker against fungi and parasites). In this study, high cell viability, upregulated expression of CD80, and downregulation of CD200R and mannose receptor CD206 was similar in LPS-stimulated MФ from all three groups showing a pro-inflammatory state similar to Jaguin et al (**Supplementary Figures 2A–D**). TLR4 surface expression was significantly higher on unstimulated preterm MФ compared to unstimulated adult MФ and downregulated on LPS-stimulated MФ (**Figure 3A**). HLA-DR surface expression was upregulated on LPS-stimulated adult MФ, but not on preterm and term MФ (**Figure 3B**). In summary, LPS stimulation induced pro-inflammatory polarization of MФ in all three groups, with a pronounced downregulation of TLR4 on preterm MФ and an upregulation of HLA-DR only on adult MФ.

#### RORC Upregulation Due to T Cell Polarization by Supernatants of Preterm MФ After LPS Stimulation

**Figure 4:**
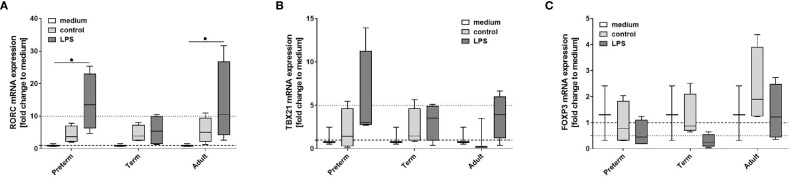
Neonatal CD4+ T cell polarization by LPS-stimulated macrophage (MФ) supernatants. Neonatal CD4+ T cells were incubated for 6 days with supernatants of LPS-stimulated preterm, term or adult MФ collected after incubation for 72 h (48 h 21% O_2_ followed by 24 h at 21% O_2_ with or without LPS). mRNA was assessed using RT qPCR. Box plots of RORC **(A)**, TBX21 **(B)** and FOXP3 **(C)** mRNA expression normalized to β-Actin depicted as a fold change to the medium control. The dotted line at 1 indicates the normalization level and the second dotted line is an auxiliary line to aid with visualization (n=4 independent 72 h supernatants were used (same as **Figure 2**) on neonatal CD4+ cells isolated from one donor, box plots: median ± 25^th^ to 75^th^ percentiles and minimum to maximum, ANOVA followed by Holm-Sidak’s test, *p<0.05).

Our data indicate that LPS-induced expression of IL-23 was unique to preterm MФ. To further evaluate the relevance of that finding, we analyzed polarization of neonatal T cells exposed to supernatants from LPS-stimulated preterm, term, and adult MФ. mRNA expression of transcription factor RORC showed greater than ten-fold upregulation in neonatal T cells exposed to supernatants from LPS-stimulated preterm and adult MФ, but not term MФ (**Figure 4A**). Furthermore, no significant differences were detected for mRNA expression of TBX21 and FoxP3 in T cells exposed to supernatants from any age group (**Figures 4B, C**). In summary, preterm and adult MФ respond to LPS stimulation with a RORC upregulation due to T cell polarization of neonatal CD4+CD25- T cells, but not term MФ.

#### Transcriptome Analysis Revealed a Global Increase of pro-Inflammatory Responses Upon LPS Stimulation in Preterm MФ Compared to Term MФ

**Figure 5:**
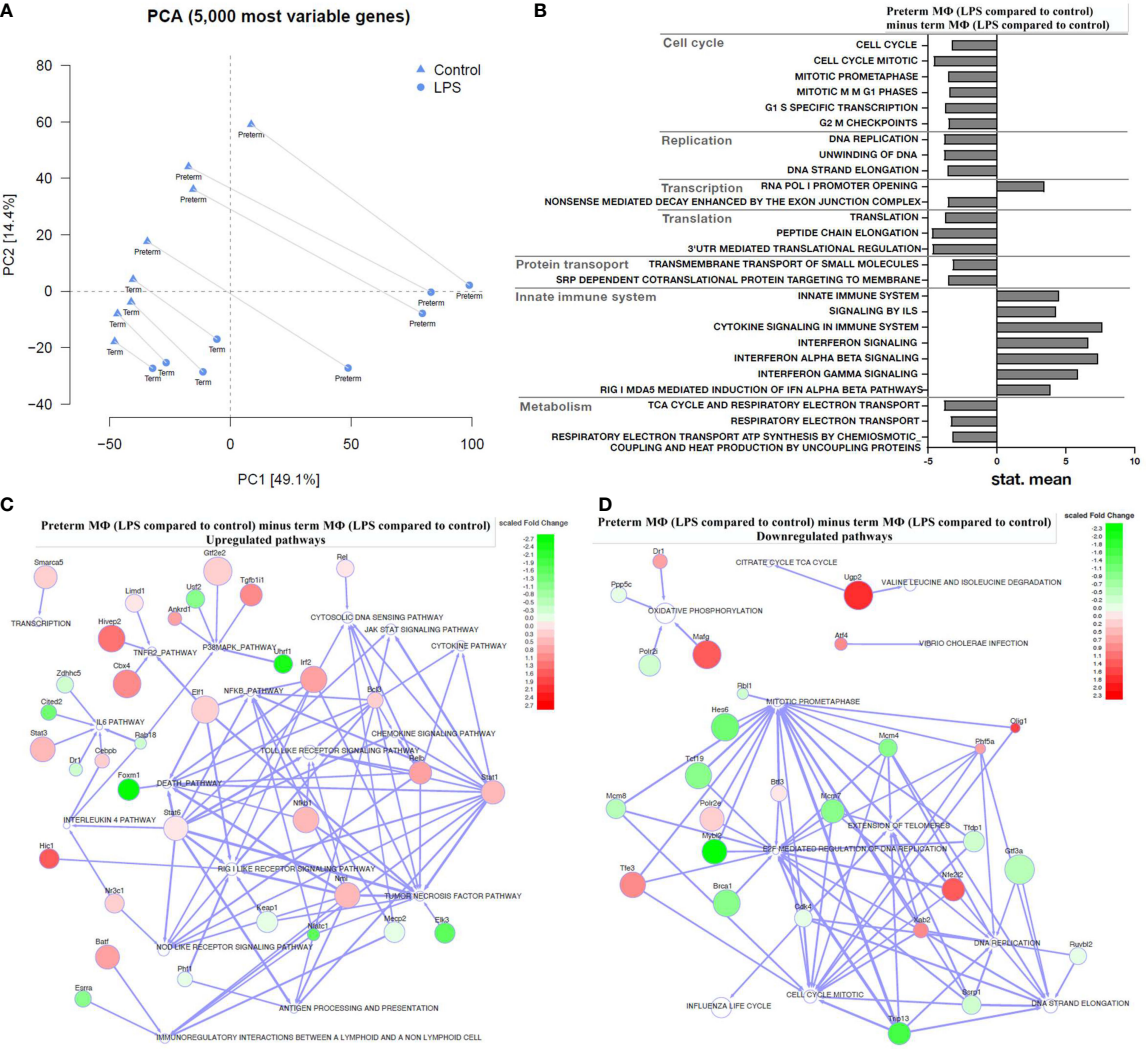
Transcriptome analysis of LPS-stimulated preterm macrophages (MΦ) compared to term MΦ. Preterm and term MФ were incubated for 72 h (48 h 21% O_2_ followed by 24 h 21% O_2_ with or without LPS). **(A)** Principal component analysis (PCA) to show distribution of samples based on the 5000 most variable genes in the RNA samples of MΦ. The PCA plot shows clusters of samples based on their similarity, which revealed clustering of RNA samples from preterm and term MΦ dependent on LPS stimulation (cluster symbols: triangles=control and circles=LPS; paired samples are connected) and gestational age (preterm *vs*. term labeling). **(B–D)** The bar graph and the regulator networks combine the effect of gestational age and stimulatory condition by comparing the difference of LPS stimulation with control (21% O_2_) from preterm MΦ to the same difference from term MΦ. **(B)** The pathway profile is based on the REACTOME gene sets (provided by MSIGDB library) after input of differentially regulated genes (unpaired t-test, p_adj_ < 0.05) from preterm and term MФ. It shows the “mean of gene set”-values which were significantly regulated. The following categories were detected: cell cycle, replication, transcription, translation, protein transport, innate immune system, and metabolism. (“mean of gene set” test statistic, p_adj_ < 0.05). **(C)** Upregulated and **(D)** downregulated pathways (white) due to significant difference in key transcriptional regulators (green: downregulated transcription factors; red: upregulated transcription factors; TRAP analysis; GAGE cutoff p<0.1; regulator cutoff p<0.01). **(A–D)**: n=4 independent experiments with cells of four different donors (3 out of 4 donors are the same as **Figure 2** 72 h time point).

To identify key differences in MФ responses to LPS stimulation on a transcriptome level, we compared the transcriptome of preterm and term MФ. As indicated in **Table 1**, the number of differentially expressed genes upon LPS stimulation was 17.4 fold higher in preterm MФ [differentially expressed genes in the LPS effect (p_adj_ < 0.05, unpaired t-test): 9,088 (4,535 up and 4,553 down)] compared to term MФ [differentially expressed genes in the LPS effect (p_adj_ < 0.05, unpaired t-test): 523 (464 up and 59 down)]. Moreover, principal component analysis (PCA) using the 5,000 most variable genes revealed a developmental separation between preterm and term MФ under unstimulated conditions, which increased even further under LPS stimulated conditions (**Figure 5A**). In addition, a cluster separation between control and LPS stimulated MФ in both groups was observed (**Figure 5A**).

**Table 1:** Numbers of differentially expressed genes in preterm and term macrophages exposed to various oxygen concentrations and LPS.

	Preterm			Term		
	Up	Down	Total	Up	Down	total
**LPS effect**						
LPS *vs*. control (21% O_2_)	4,535	4,553	9,088	464	59	523
**Oxygen effect**						
65% O_2_ *vs*. control	16	14	30	13	15	28
3% O_2_ *vs*. control	0	0	0	1	1	2
**Double-hit effect**
65% O_2_ + LPS *vs*. LPS	39	68	107	63	232	295
3% O_2_ + LPS *vs*. LPS	0	0	0	0	2	2

For priming with 65% O_2_ or 3% O_2_ followed by LPS stimulation, preterm and term MФ were incubated for 72 h (48 h 21%, 65% or 3% O_2_ followed by 24 h 21% O_2_ with or without LPS). (n=4 independent experiments with cells of four different donors (same as **Figures 5** and **9**); p_adj_ < 0.05, unpaired t-test).

In the following results, gene set enrichment analysis against the MUELLER PLURINET gene set, the REACTOME gene sets and the transcriptional regulatory associations in pathways (TRAP) analysis were performed on log2 fold changes using GAGE (v2.32.0). We found no significant differences of gene expression in unstimulated preterm MФ compared to unstimulated term MФ using a gene network specific for cell differentiation (p=0.1240; genes constituting the PLURINET protein-protein network shared by pluripotent cells). This result was confirmed analyzing the pathway profile using the REACTOME database which did not detect differences between unstimulated preterm MФ compared to unstimulated term MФ (cut-off p_adj_ < 0.05).

Next, a transcriptome pathway profile using the REACTOME database was performed by calculating the LPS effects in preterm MФ minus LPS effects in term MФ (see **Figure 1** for LPS effect definition). The pathway profile revealed a more pronounced upregulation of pathways belonging to the innate immune system (Signaling by Interleukins, Cytokine Signaling, Interferon Signaling) in LPS-stimulated preterm MФ at 72 h compared to term MФ (LPS effect in each age group not shown, LPS effect in preterm MФ minus LPS effect in term MФ see **Figure 5B**). In addition, pathways belonging to cell cycle and cell functions such as replication, transcription, translation, and transport had a more accentuated downregulation in preterm as compared to term MФ upon LPS stimulation. Furthermore, metabolic pathways such as tricarboxylic acid (TCA) cycle and respiratory electron transport were only downregulated in LPS-stimulated preterm MФ compared to term MФ (LPS effect in each age group not shown, LPS effect in preterm MФ minus LPS effect in term MФ see **Figure 5B**).

Afterwards, a TRAP analysis was performed by calculating the LPS effects in preterm MФ minus LPS effects in term MФ (see **Figure 1** for LPS effect definition) to identify gene-pathway transcriptional regulatory relationships. The resulting TRAP network (**Figures 5C, D**) therefore visualizes the most significant regulator-pathway associations existing within the expression profile of preterm MФ. All 62 significantly up- and downregulated key transcriptional regulators in LPS-stimulated preterm MФ compared to term MФ are depicted in **Supplementary Figure 3** (Regulator cutoff p_adj_ < 0.01). The strongest downregulation of transcriptional regulators was found in Foxm1, Uhrf1, Nfatc1, Elk3, Cited2, and Usf2. As a consequence, certain pathways such as death pathway, p38MAPK, NFκB, tumor necrosis factor (TNF), and IL6 signaling are upregulated (**Figure 5C**). The strongest upregulation of transcriptional regulators was found in Cbx4, Tgfb1i1, Hivep2, and Hic1 which induces upregulation of TNFR2, p38MAPK, and RIG I like receptor signaling pathways (**Figure 5C**). Downregulated pathways such as cell cycle mitotic, DNA replication, oxidative phosphorylation, and TCA cycle were mainly related to downregulated expression of Mybl2, Trip13, and Hes6 and upregulated expression of Mafg, Nfe2l2, Olig1, and Ugp2 (**Figure 5D**).

This analysis confirmed a stronger upregulation of innate immune pathways such as NFκB signaling, Jak-STAT signaling, NOD-like receptor signaling and TLR signaling in LPS-stimulated preterm MФ compared to term MФ (**Figure 5C**). In addition, some specific cytokine pathways (e.g. IL-12, IL-6, and TNF signaling) were shown to be upregulated by key transcriptional regulators in LPS-stimulated preterm MФ compared to term MФ (**Figure 5C**). Other signaling pathways related to cell functions (e.g. replication) and metabolic signaling (e.g. TCA cycle) were confirmed to be downregulated (**Figure 5B, D**). In summary, a globally enhanced pro-inflammatory phenotype of LPS-stimulated preterm MФ was detected on transcriptome level while unstimulated MФ showed no differences in expression patterns between age groups.

### Effects of Priming With Hyperoxia or Hypoxia on the Immune Response of Preterm MΦ Upon LPS Stimulation

To determine whether preterm MФ respond differently after exposure to multiple factors, we assayed responses of preterm, term, and adult MФ exposed to different O_2_ conditions and subsequent LPS stimulation (**Figure 1**). This double-hit model was developed to mimic key factors previously shown to increase risk for BPD in preterm infants, including oxygen concentration (65% O_2_ to model the need of supplemental O_2_, 3% O_2_ to mimic hypoxic episodes, and 21% O_2_ as a control) followed by LPS stimulation as a surrogate for infection. As above, we investigated cytokine release, activation marker expression, neonatal T helper polarization, and transcriptome analysis of MФ. We verified O_2_ sensing by neonatal MФ (monocytes isolated from term infants and subsequently differentiated to MФ). Nuclear factor-like (Nrf)2 protein, which is stabilized upon oxidative stress, increased in neonatal MФ exposed to 65% O_2_, whereas hypoxia inducible factor (HIF)-1α, which is usually stabilized upon hypoxic conditions, was upregulated upon incubation of neonatal MФ in 3% O_2_ (**Supplementary Methods 3** and **Supplementary Figure 4**).

#### Opposing Effects of Priming With Hyperoxia or Hypoxia on Preterm MФ Cytokine Responses

**Figure 6:**
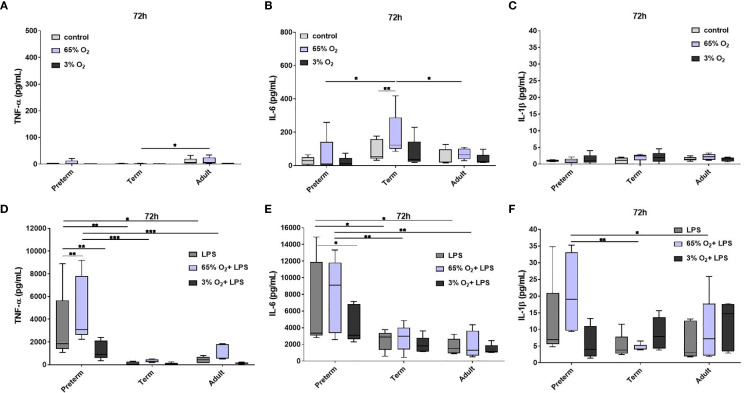
Pro-inflammatory cytokine release in preterm, term and adult macrophages (MФ) primed with various O_2_ concentrations followed by LPS. TNFα, IL-6, and IL-1β release after O_2_ exposure only compared to control (21% O_2_) **(A–C)** and after priming with 65% O_2_ or 3% O_2_ followed by LPS compared to LPS alone **(D–F)** incubated for 72 h (48 h 65% O_2_ or 3% O_2_ followed by 24 h 21% O_2_ with or without LPS). Cytokine release was measured in the supernatant of all three groups using a cytokine bead array (n=5 independent experiments with supernatants of five different donors (same donors as **Figure 2** 72 h time point; control and LPS values were re-used from **Figure 2** because oxygen and double-hit conditions were included in those experiments), box plots: median ± 25^th^ to 75^th^ percentiles and minimum to maximum, ANOVA followed by Holm-Sidak’s test, *p<0.05, **p<0.01, and ***p<0.001).

We evaluated cytokine release from MФ exposed to different O_2_ concentrations followed by LPS stimulation. Using the control conditions 65% and 3% O_2_ without LPS stimulation, cytokine release showed minor differences predominantly in term MΦ, however cytokine concentrations are generally much lower than in the LPS stimulated MΦ (**Figures 6A–C** and **Supplementary Figure 5A, B**). Therefore, we focused on the changes upon priming with 65% O_2_ or 3% O_2_ and subsequent LPS stimulation in comparison to the LPS condition alone to determine additional effects caused by double-hit exposures. After 72 h incubation, we observed a further increase of TNFα, and the same trend for IL-6 and IL-1β upon 65% O_2_ and subsequent LPS stimulation of preterm MΦ, which was not detected in term and adult MΦ (**Figures 6D–F**). All other cytokines (IL-10, IL-12p40 and IL-23) were similarly expressed compared to patterns of LPS stimulated MΦ (**Supplementary Figure 5C, D**). IL12p70 and IFNγ release were still not detected in any group. An opposing effect was detected after priming with 3% O_2_ where the subsequent LPS stimulation leads to a decreased cytokine expression pattern for TNFα, IL-6 and IL-1β (**Figures 6D–F**).

In summary, the cytokine release of preterm MФ after priming with 65% O_2_ and subsequent LPS stimulation indicated an exaggerated pro-inflammatory profile compared to term and adult MФ whereas priming the preterm MФ with 3% O_2_ leads to a slight suppression of pro-inflammatory cytokine release.

#### Oxygen Exposure Mainly Affects Activation Marker Expression on LPS-Stimulated Preterm MФ

**Figure 7:**
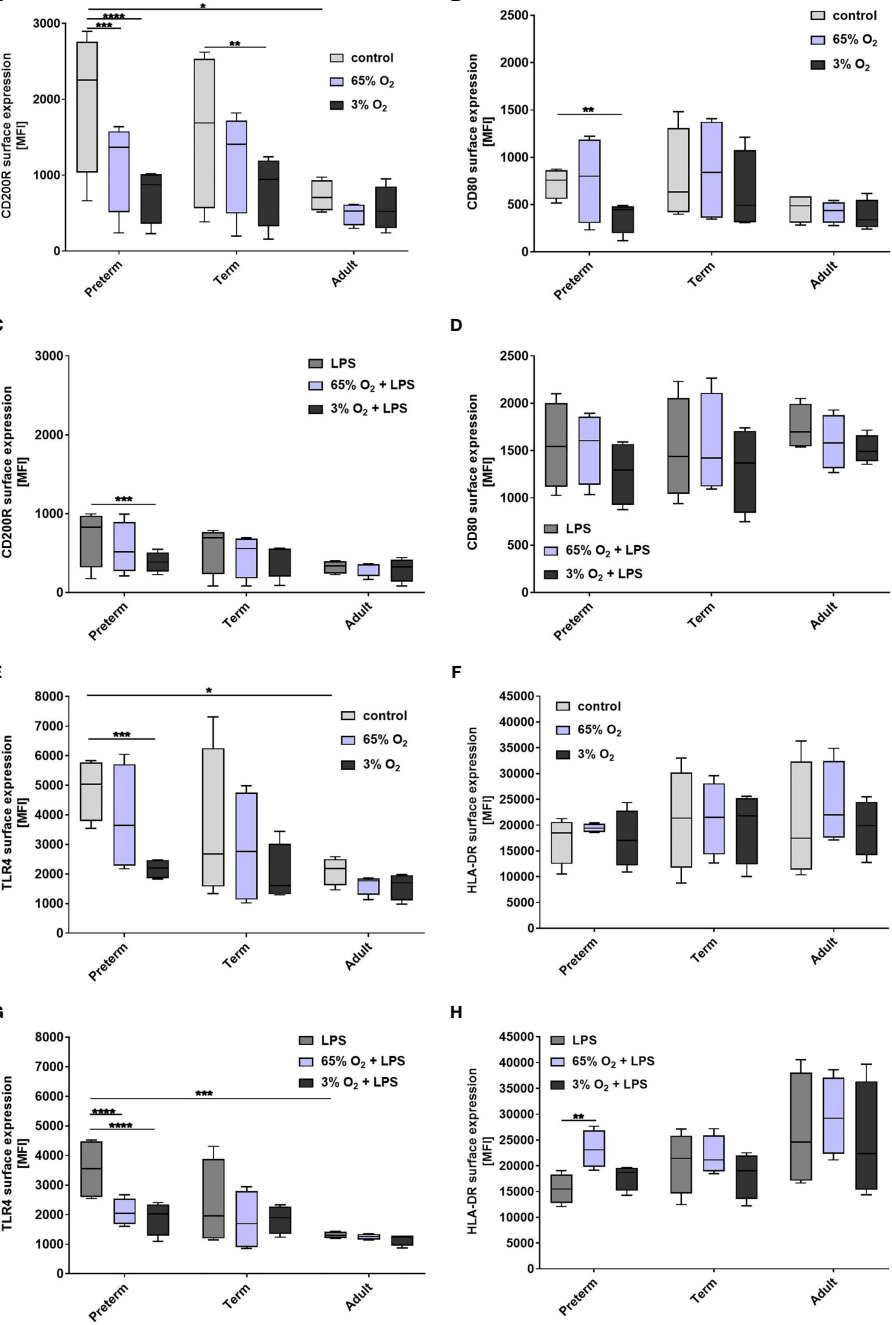
Expression of activation marker on preterm, term, and adult macrophages (MФ) primed with various O_2_ concentrations followed by LPS. Surface expression of CD200R, CD80, TLR4, and HLA-DR after O_2_ exposure only compared to control (21% O_2_) **(A, B, E, F)** and after priming with 65% O_2_ or 3% O_2_ followed by LPS compared to LPS alone **(C, D, G, H)** incubated for 72 h (48 h 65% O_2_ or 3% O_2_ followed by 24 h 21% O_2_ with or without LPS). Mean fluorescence intensity [MFI] of surface proteins is shown on macrophages in all three groups assessed by flow cytometry. (n=4 independent experiments with cells of four different donors (same donors as **Figure 3** ; control and LPS values were re-used from **Figure 3** because oxygen and double-hit conditions were included in those experiments), box plots: median ± 25^th^ to 75^th^ percentiles and minimum to maximum, ANOVA followed by Holm-Sidak’s test, *p < 0.05, **p < 0.01, ***p < 0.001, and ****p < 0.0001).

To characterize MФ activation after exposure to various O_2_ concentrations and LPS, we analyzed surface expression of CD80, CD200R, CD206, TLR4 and HLA-DR. Due to the distinct pro-inflammatory cytokine profiles of preterm MФ after priming with 65% O_2_ or 3% O_2_ and subsequent LPS, we hypothesized that patterns of activation of pro-/and anti-inflammatory markers differ remarkably between age groups. Priming with 65% O_2_ alone downregulated regulatory CD200R expression on preterm MФ compared to unstimulated MФ, and had no additional effect on any marker (**Figures 7A, B, E, F**). Combining priming with 65% O_2_ and subsequent LPS led to a further downregulation of TLR4 surface expression and an upregulation of HLA-DR surface expression only on preterm MФ compared to control preterm MΦ stimulated with LPS (**Figures 7G, H**). Priming with 3% O_2_ alone suppressed CD200R, CD80 and TLR4 surface expression on preterm MФ compared to unstimulated MФ (**Figures 7A, B, E, F**). Priming with 3% O_2_ led to a further downregulation of regulatory CD200R and TLR4 surface expression on LPS-stimulated preterm MФ (**Figures 7C, D, G, H**). Priming with different O_2_ concentrations alone or with additional subsequent LPS stimulation did not affect any activation marker expression on term and adult MФ, except for downregulation of regulatory CD200R on term MФ upon priming with 3% O_2_ alone (**Figure 7A**). Likewise, O_2_ concentration did not affect CD206 expression on preterm, term or adult MФ stimulated with LPS (data not shown). Of note, the mean viability of MФ upon double-hit stimulation with either 65% or 3% O_2_ alone and in combination with LPS was always above 90% which is similar to LPS stimulation alone in all three groups (data not shown). In summary, both priming with hypoxia or hyperoxia downregulated TLR4 expression and regulatory CD200R expression in preterm MФ while 65% O_2_ concentration also enhanced LPS-induced HLA-DR expression. In contrast, O_2_ concentration did not affect the expression of TLR4 and HLA-DR activation markers on term or adult MФ.

#### Effects of Macrophage Exposure to Oxygen and LPS on FoxP3 Expression in T Cell Polarization

**Figure 8:**
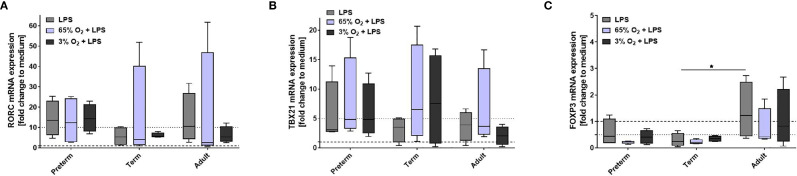
Neonatal CD4+ T cell polarization by supernatants of macrophages (MФ) primed with various O_2_ concentrations followed by LPS. Neonatal CD4+ T cells were incubated for 6 days with supernatants of preterm, term or adult MФ after priming MФ with 65% O_2_ or 3% O_2_ followed by LPS compared to LPS alone. Supernatants were collected after incubation of MФ for 72 h (48 h 65% O_2_ or 3% O_2_ followed by 24 h 21% O_2_ with or without LPS). mRNA was assessed using RT qPCR. Box plots of RORC **(A)**, TBX21 **(B)** and FOXP3 **(C)** mRNA expression normalized to β-Actin depicted as a fold change to the medium control. The dotted line at 1 indicates the normalization level and the second dotted line is an auxiliary line for visualization (n=4 independent 72 h supernatants were used (same as **Figure 6**) on neonatal CD4+ cells isolated from one donor, LPS values were re-used from **Figure 4** because double-hit conditions were included in those experiments, box plots: median ± 25^th^ to 75^th^ percentiles and minimum to maximum, ANOVA followed by Holm-Sidak’s test, *p < 0.05).

Next, we characterized the polarization of neonatal T cells induced by supernatants from MФ exposed to various oxygen concentrations and stimulated with LPS. T cell mRNA expression of RORC, TBX21 and FoxP3 following exposure to supernatants of MФ that were not stimulated with LPS was not affected by O_2_ concentrations in which MФ were cultured (data not shown). Furthermore, no significant differences were detected for mRNA expression of RORC and TBX21 (**Figures 8A, B**). In general, there was a lower FoxP3 mRNA expression in neonatal T cells exposed to supernatants from LPS-stimulated term and preterm MФ compared to adult MФ, but there were no significant differences based on O_2_ concentration (**Figure 8C**). In summary, FoxP3 expression due to T cell polarization is decreased by term and preterm MФ upon pro-inflammatory conditions compared to adult MФ.

#### Oxygen Exposure Leads to More Pronounced Upregulation of Pro-Inflammatory Pathway Profiles in LPS-Stimulated Preterm Macrophages

To identify the main differences in the immune response on transcriptome level after priming the MФ with hyperoxia or hypoxia, we compared the transcriptome of LPS-stimulated preterm and term MФ exposed to various O_2_ concentrations. The lack of strong immune responses in MФ after stimulation with 65% or 3% O_2_ alone compared to the unstimulated control was confirmed in the transcriptome analysis, with relatively few differentially expressed genes in the oxygen effect for preterm and term MФ (**Table 1**: maximum of 30 genes differentially expressed in the 65% O_2_ *vs.* control condition). In the following, gene set enrichment analyses against the MUELLER PLURINET gene set and the REACTOME gene sets were performed again using GAGE (v2.32.0). A significant difference of gene expression for priming with 65% O_2_ *versus* unstimulated control (21% O_2_) was detected in preterm and term MФ using a gene network specific for cell differentiation (Term: p=1.35909x10^-12^; Preterm: p = 8.037793x10^-10^; genes constituting the PLURINET protein-protein network shared by pluripotent cells); this effect was not observed for 3% O_2_. However, analyzing the pathway profile using the REACTOME database did not reveal significantly changed pathways in the oxygen conditions alone compared to unstimulated control in preterm and term MФ (cut-off p_adj_ < 0.05).

The number of differentially expressed genes upon LPS stimulation was greater in preterm and term MФ primed with 65% O_2_ compared to control conditions (65% O_2_ + LPS *vs.* LPS (p_adj_ < 0.05, unpaired t-test): total of 107 differentially expressed genes in preterm MФ and 295 in term MФ). In contrast, priming with 3% O_2_ had a minimal effect on the number of differentially expressed genes in LPS-stimulated MФ (**Table 1**).

**Figure 9:**
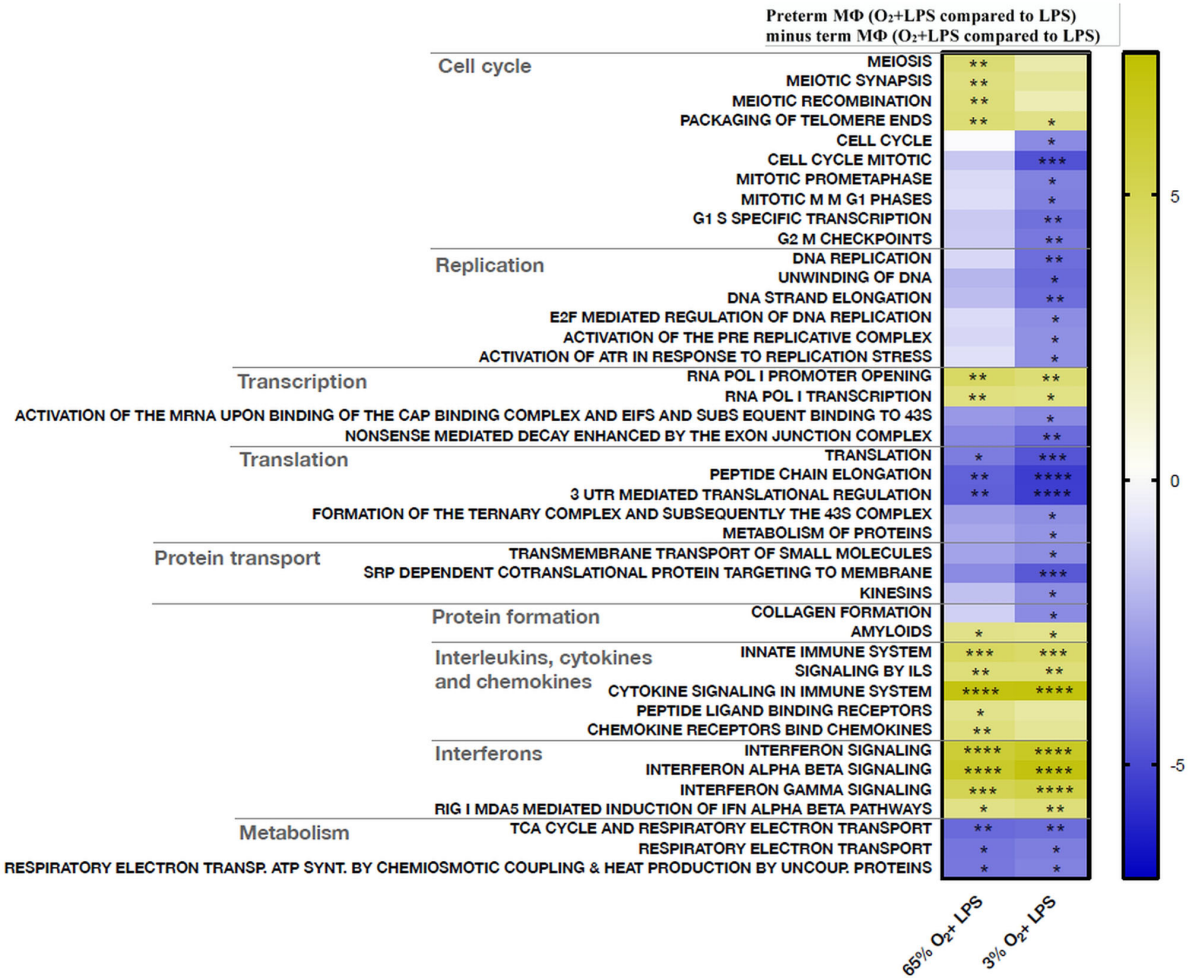
Transcriptome pathway profile of preterm macrophages (MΦ) exposed to various oxygen concentrations and LPS compared to term MΦ. For priming with 65% O_2_ or 3% O_2_ followed by LPS stimulation, preterm and term MФ were incubated for 72 h (48 h 65% O_2_ or 3% O_2_ followed by 24 h 21% O_2_ with or without LPS). The heatmap compares the responsiveness to oxygen priming between different gestational age groups by comparing the difference of O_2_+LPS with LPS alone from preterm MΦ subtracted by the same difference from term MΦ (LPS values were re-used from because double-hit conditions were included in those experiments). The pathway profile is based on the REACTOME gene sets (provided by MSIGDB library) after input of differentially regulated genes (p_adj_ < 0.05) from preterm and term MФ. It shows the “mean of gene set”-values which were significantly regulated in at least one double-hit condition *vs.* LPS indicated be an asteriks. The following categories were detected: cell cycle, replication, transcription, translation, protein transport and formation, innate immune system, and metabolism. (n=4 independent experiments with cells of four different donors (3 out of 4 donors are the same as **Figure 6**); “mean of gene set” test statistic, *p<0.05, **p<0.01, ***p<0.001, and ****p<0.0001).

Next, a transcriptome pathway profile using the REACTOME database was performed by comparing oxygen-primed LPS stimulation with LPS alone within the groups. This comparison led to a significant downregulation of pathways belonging to the cell cycle, replication, transcription, and translation in term MΦ. However, no differences were detected in pathway signaling of oxygen-primed LPS-stimulated preterm MΦ compared to LPS stimulation alone (data not shown). Therefore, we analyzed the double-hit effect in preterm MФ minus the double-hit effect in term MФ (see **Figure 1** for Double-hit effect definition). Hence, the heatmap compares the responsiveness to oxygen priming between different gestational age groups by comparing the difference of O_2_+LPS with LPS alone from preterm MΦ subtracted by the same difference from term MΦ (**Figure 9**). The pathway profile revealed that the response to priming with either 65% or 3% O_2_ led to a more pronounced upregulation of pathways belonging to the innate immune system (Signaling by Interleukins, Cytokine Signaling, Interferon Signaling) in LPS stimulated preterm MФ compared to the response of LPS-stimulated term MФ (**Figure 9**, lower part of heatmap). However, the pathway “chemokine receptors bind chemokines” was only more upregulated upon priming with 65% O_2_, but not 3% O_2_ in LPS-stimulated preterm MΦ compared to the response of LPS-stimulated term MФ. In addition, metabolic pathways were more downregulated in response to priming with 65% O_2_ and 3% O_2_ in LPS-stimulated preterm MΦ compared to the same response in term MФ (**Figure 9**, last three pathways). Furthermore, a more pronounced downregulation of pathways belonging to cell cycle and cell functions such as replication, transcription, translation, and transport was only observed upon priming with 3% O_2_ in LPS-stimulated preterm MΦ compared to the same response in term MΦ (**Figure 9**, upper part of the heatmap). After priming with 65% O_2_, we rather detected an upregulation of pathways belonging to the cell cycle (**Figure 9**, first three pathways). In summary, the pathway profile of LPS-stimulated preterm MФ revealed a stronger pro-inflammatory response to oxygen priming on transcriptome level than LPS-stimulated term MФ.

#### Oxygen Exposure Contributes to Sustained Inflammatory Responses in Preterm MФ Through Downregulation of Egr2 and Gfi1 Transcription Factors

We used TRAP analysis to identify key transcriptional regulators and the affected pathways in MФ exposed to various O_2_ concentrations and LPS. In the following results, the transcriptional regulatory associations in pathways (TRAP) analysis were performed again using GAGE (v2.32.0). Here, we compared LPS-stimulated preterm MФ primed with 65% O_2_ or 3% O_2_ to control conditions (LPS alone) with the same stimulatory conditions of term MФ (double-hit effect in preterm MФ minus double-hit effect in term MФ; see **Figure 1** for Double-hit effect definition). 25 of 62 transcriptional regulators affected by LPS exposure alone were further regulated when preterm MФ were primed with 65% or 3% O_2_ (**Supplementary Figure 3**: green bars). Additional 25 transcriptional regulators affected by LPS exposure alone were further regulated when preterm MФ were primed with 3% O_2_ compared to the same stimulatory conditions of term MФ (**Supplementary Figure 3**: blue bars). The latter transcriptional regulators invoked a further increase of innate immune pathways such as NFκB pathway (e.g by Nfatc1 downregulation) as well as a further decrease of metabolic and cell function pathways such as oxidative phosphorylation (e.g., by Mafg upregulation) compared to LPS stimulation alone in preterm MФ. Specific transcriptional regulators that were only affected in LPS-stimulated preterm MФ primed with 65% and 3% O_2_ are shown in **Table 2**. Egr2 and Gfi1 showed the most prominent effects after priming with 65% O_2_ and 3% O_2_, respectively, in LPS-stimulated preterm MФ. Interestingly, a differential effect (downregulation) on the early growth response gene (Egr)-2 was only detected in LPS-stimulated preterm MФ primed with 65% O_2_ compared to the same stimulatory condition of term MФ. Furthermore, only priming with 3% O_2_ led to a downregulation of growth factor independence 1 (Gfi1) in LPS-stimulated preterm MФ compared to the same stimulatory condition of term MФ.

**Table 2:** Key transcriptional regulators significantly regulated in preterm macrophages exposed to various oxygen concentrations and LPS compared to term MΦ.

Preterm MФ (O_2_+LPS compared to LPS) minus term MФ (O_2_+LPS compared to LPS)		
Comparison	Gene name	logFC
**65%/3% double-hits *vs.* LPS**	Ets1	0.2368/0.4940
**65% Double-hit *vs.* LPS**	**Egr2**	**-2.2528**
	Arhgab17	-1.0469
	Ptges2	-0.4509
	Khdrbs1	-0.3903
**3% Double-hit *vs.* LPS**	**Gfi1**	**-1.9619**
	Pdcd11	-0.1357
	Ikbkb	-0.1446
	Zfx	0.0658
	Tceal1	0.0203
	Nr1d2	0.0293

For priming with 65% O_2_ or 3% O_2_ followed by LPS stimulation, preterm and term MФ were incubated for 72 h (48 h 65% or 3% O_2_ followed by 24 h 21% O_2_ with or without LPS). Regulators were analyzed by combining the effect of gestational age and stimulatory condition. The effect difference of double-hit conditions with LPS alone in preterm MΦ compared to the respective difference in term MΦ. Additional 50 of 62 key transcriptional regulators, which already appeared in LPS vs control, are further regulated in the double-hit effects (**Supplementary Figure 3**: bars depicted in blue and green). (n=4 independent experiments with cells of four different donors (same as **Figure 9**); p_adj_ < 0.01; logFC refers to estimated log2 fold-changes).

Bold: means the strongest of all values in that condition.

In summary, the transcriptome analysis supported an exaggerated pro-inflammatory immune response in preterm MФ compared to term MФ following priming with a combination of hyperoxia or hypoxia and subsequent LPS stimulation. Additional analysis suggested that differences in key transcriptional regulators (Egr2: priming with 65% O_2_; Gfi1: priming with 3% O_2_) may contribute to those effects.

## Discussion

To our knowledge, this is the first explorative study investigating human monocyte-derived MФ of preterm infants in a double-hit model. We noted that MФ of preterm infants have an enhanced and sustained pro-inflammatory response to LPS compared to MФ of term infants and adults, and that preterm MФ responses induce RORC upregulation due to neonatal T cell polarization in neonatal T cells. In addition, the enhanced and sustained pro-inflammatory response of preterm MФ was exaggerated after exposure to key factors (hyperoxia and hypoxia followed by LPS stimulation) that are strongly associated with the development of BPD in a clinical setting. The response was characterized by enhanced pro-inflammatory cytokine release, increased HLA-DR surface expression and downregulated FoxP3 expression due to neonatal T cell polarization. The transcriptome pathway profiling of preterm MФ confirmed those findings and revealed a possible role for downregulation of the transcriptional regulators Egr2 and Gfi1 in excessive pro-inflammatory MФ polarization of preterm infants.

We hypothesized gestational age-dependent differences in MФ. The MФ phenotype was similar across gestational ages after differentiation with M-CSF with regard to morphology, viability and the phenotypic markers CD14, CD68, and CD11b (**Supplementary Figure 1**), suggesting that monocytes from preterm infants are responsive to M-CSF. After differentiation, we stimulated preterm, term and adult MФ with LPS to investigate gestational age-dependent differences in their immune response. Generally, the cytokine expression patterns of preterm MФ suggested a robust TLR-mediated response, which was even enhanced for specific mediators as compared to the response of term and adult MФ. The response of preterm MФ was consistent with previous data supporting Th17-type immunity in neonatal monocytes (2, 4, 35). Interestingly, prolonged LPS stimulation (72 h, compared to 52 h) led to sustained release levels of pro-inflammatory TNFα, IL-6 and IL-1β and an enhancement of IL-23 and IL-10 release (**Figure 2**). That effect was less pronounced for term and adult MФ. Anti-inflammatory IL-10 is probably a regulatory mechanism of the extensive pro-inflammatory cytokine release of preterm MФ. The excessive IL-10 release from preterm MФ might function to prevent TNFα-induced cell death due to IL-10-mediated shedding of TNFR2 for neutralization of soluble TNF (36). This hypothesis is supported by the upregulation of the TNFR2 pathway in the TRAP analysis of the transcriptome (**Figure 5C**). However, excessive IL-10 release could also have a negative effect, especially in the context of lung inflammation. Animal experiments have shown that pulmonary fibrosis worsens with over expression of IL-10 and development of fibrotic tissue is consistent with long-term sustained lung inflammation (37).

The mechanism for enhanced pro-inflammatory cytokine release by preterm MΦ is proposed to be mediated by TLR4 signaling due to increased basal TLR4 expression (**Figure 3**: control). Other studies have analyzed cytokine production and TLR4 expression in preterm monocytes. Levels of cytokine production and TLR4 expression by monocytes were shown to be positively correlated with gestational age (28, 38). Those correlations suggest that TLR4 signaling might contribute to the detected cytokine levels in monocytes of preterm infants. In this study, preterm MФ demonstrated increased basal TLR4 expression and sustained cytokine production compared to term and adult MФ, therefore it seems likely that TLR4 is part of the mechanism for the produced cytokine amounts. The decreased TLR4 surface expression upon LPS stimulation might be due to a negative feedback regulation to strong pro-inflammatory responses (39).

Supernatant of preterm MФ, but not term MФ, increased the expression of RORC mRNA in neonatal CD4+CD25- T cells (**Figure 4**). This could suggest a Th17 polarization. Monocytes isolated from preterm infants who developed necrotizing enterocolitis have also been shown to preferentially support Th17 polarization as opposed to Treg polarization (31). Similarly, in a neonatal mouse model, necrotizing enterocolitis-induced systemic inflammation leading to lung injury was mediated by Th17 cells in the lung (40). However, in cord blood of term infants, a small subset of CD45RO+CCR7-CD25low CCR6+ effector memory T cells (TEM) has a profile of pro-Th17 cells, expressing RORC (41). These cells also produce IL-17 if they are activated in the presence of IL-1β and IL-23. It is therefore possible that the pro- inflammatory cytokine profile in the supernatants of macrophages could activate or expand these pro-Th17 cells.

The transcriptome pathway analysis of preterm and term MΦ stimulated with LPS confirmed gestational-age dependent differences, with upregulation of pro-inflammatory immune pathways and downregulation of pathways supporting the cell cycle and basic cell functions in preterm MΦ compared to term MΦ (**Figures 5A, B**). In particular, TRAP analysis revealed effects on key transcriptional regulators (e.g. Nfatc1, Elk3, Cbx4 and Cited2) and specific upregulation of pro-inflammatory pathways such as NFκB, TLR signaling, TNF, and IL-6 signaling in LPS-stimulated preterm MΦ compared to term MΦ (**Figures 5C, D**, **Supplementary Figure 3**). Furthermore, Ugp2 and Mafg were upregulated transcription factors leading to TCA cycle and respiratory electron transport pathway downregulation in preterm MΦ compared to term MΦ upon LPS stimulation (**Figures 5C, D**, **Supplementary Figure 3**). The effect on these metabolism pathways provides additional support for the concept of a pro-inflammatory activated preterm MΦ phenotype, because downregulation of these pathways indicates a switch in energy metabolism from oxidative phosphorylation to glycolysis, which has been described for pro-inflammatory MФ (42, 43). However, another study suggests a deficient glycolytic metabolism in term MΦ (25). Therefore, further functional investigation of the metabolism of preterm MΦ is needed.

Since LPS-stimulated preterm MΦ showed an enhanced pro-inflammatory phenotype compared to term MΦ, we investigated differences between the unstimulated condition of preterm and term MΦ on transcriptome level. However, no difference in cell differentiation genes was detected between the unstimulated conditions of preterm and term MΦ. Therefore, the origin of an enhanced LPS-stimulated pro-inflammatory preterm MΦ phenotype needs further investigation. One could speculate that the different immune response of preterm and term MΦ might be due to an altered DNA methylation pattern in preterm MΦ. Another study has shown that cord blood hematopoietic cells from preterm infants display altered DNA methylation patterns compared to term infants (44).

Human MФ are a key immune cell population in lung diseases of preterm infants. For the first time, we modeled key lung exposure factors such as different O_2_ conditions and LPS in human preterm MФ. Some studies have demonstrated that the double-hit exposure can change the immune response (18–20) and that this is especially important in the context of a multifactorial disease such as BPD. In a mouse model, the combination of high O_2_ concentration and LPS exposure was associated with a detrimental immune response characterized by an impaired type 2 anti-inflammatory environment and changes in the MФ populations of the lung leading to developmental arrest (20). The use of primary human MΦ from preterm infants, in contrast to animal models, has the advantage of providing deeper insight into functions of specific human immune cells. In addition, MФ are especially important for local immune processes within tissues, such as mucosal-associated lung and gut tissues (3). Here, we report an exaggerated pro-inflammatory immune response exclusively in preterm MФ upon stimulation with different O_2_ conditions and sequential LPS stimulation *versus* LPS stimulation alone. The exaggerated effect primed by 65% O_2_ conditions is characterized by increased pro-inflammatory cytokines (**Figure 6**), increased HLA-DR surface expression, and decreased TLR4 surface expression (**Figure 7**) as well as downregulated FoxP3 expression due to T cell polarization of neonatal CD4+CD25- cells (**Figure 8**). The transcriptome pathway profile of preterm MФ confirmed an exaggerated pro-inflammatory immune response when primed with high O_2_ conditions and sequential LPS stimulation, with enhanced responsiveness of immune pathways including a more pronounced upregulation of the chemokine pathways and stronger downregulation of TCA cycle and respiratory electron transport pathways (**Figure 9**). Another study also showed an exaggerated pro-inflammatory signature in preterm lung MФ from BPD patients using transcriptome analysis (45). Using TRAP analysis, we identified downregulation of the transcriptional regulator Egr2 in preterm MФ upon priming with 65% O_2_ followed by LPS stimulation (**Table 2**). Egr2 is described to be involved in anti-inflammatory MФ polarization (46), and its downregulation might be the driving mechanism for an excessive pro-inflammatory response of preterm MФ after priming with 65% O_2_. Detection of this mechanism might provide an early target for preventive strategies against the development of sustained inflammation in the lung.

We observed similar changes in preterm MФ exposed to 3% O_2_ and subsequent LPS stimulation, including CD200R and TLR4 surface expression decrease (**Figure 7**), and a downregulation of FoxP3 expression due to T cell polarization of CD4+CD25- cells (**Figure 8**), but cytokine release was rather downregulated after priming with 3% O2 (**Figure 6**). Transcriptome pathway analysis, however, revealed a greater responsiveness with a more pronounced upregulation of immune pathways and downregulation of TCA cycle and respiratory electron transport pathways in preterm MФ exposed to 3% O2 and LPS similar to 65% O_2_ priming (**Figure 9**), suggesting common effects of both hypoxia and hyperoxia at the gene expression level. This is in line with previous observations that hypoxia induces a pro-inflammatory immune response due to the activation of NFκB by hypoxia-inducible transcription factor (HIF) (47). As potential mechanism, we propose downregulation of Gfi1 in preterm MФ upon priming with 3% O_2_ and sequential LPS stimulation. In MФ, Gfi1 has been described to inhibit pro-inflammatory cytokines after exposure to gram-negative bacteria or to LPS (48). Uncontrolled release of these cytokines in Gfi1-null mice lead to sepsis and rapid death from infection (48). Hence, our data on Gfi-1 downregulation after priming with 3% O_2_ provide the basis for future preventive targets against the development of sustained inflammation in the lung.

Since an extensive pro-inflammatory response was observed only in LPS-stimulated preterm MФ after priming with both high and low O_2_ conditions compared to term MФ, the control conditions using high and low O_2_ conditions alone were included to investigate the differences upon priming of preterm and term MФ. Using the transcriptome dataset, a significant difference of gene expression in 65% O_2_ *versus* unstimulated control (21% O_2_) was detected using a gene network specific for cell differentiation both in preterm and term MФ. No differences were found for comparisons of 3% O_2_ *vs.* 21% O_2_. Therefore, the origin of an exaggerated pro-inflammatory preterm MΦ phenotype due to priming with various O_2_ conditions needs further investigation. Priming with O_2_ might also trigger an altered DNA methylation as described before for the use of supplemental oxygen in preterm infants after birth (49).

MФ isolated from preterm infants provide a useful model to study immune responses related to lung immunity due to their tissue-specific abundance (50) and dual involvement in tissue development and lung immunity (21). In addition, a similar exaggerated pro-inflammatory transcriptome signature in preterm lung MФ from BPD patients has been demonstrated (45) and several animal models for BPD reported recruitment of macrophages to the lung likely due to monocyte influx from the blood (20, 51). However, it needs to be considered that this model might not account for functional differences in tissue resident lung MФ and for environmental factors in the context of lung tissue *in vivo*, which might influence important MФ responses. Lung organoid models might provide a useful tool to study preterm MФ in the context of lung tissue more closely related to the environment in the infant lung. It should also be noted that monocytes from preterm and term infants were obtained from umbilical cord blood to facilitate the collection of sufficient numbers from infants. There is, however, the potential for a small amount of contamination with maternal blood during clinical sampling as well as the possibility of a different immune response compared to peripheral blood cells. Future studies need to consider peripheral blood of infants and laboratory methods adjusted to use minimal-volume samples because inflammatory responses rapidly mature after birth even in extremely preterm infants (52). Despite these limitations, primary MФ from preterm infants provide a useful *ex-vivo* model for studying immune responses in a context that is relevant to highly vulnerable infants. The double-hit model used in this study simulates exposure to two important factors that are often encountered by preterm infants in the first weeks of life (5, 6). However, it needs to be taken into account that preterm infants often have fluctuating oxygen levels, with phases of hypoxia due to apnoea alternating with phases of hyperoxia due to oxygen supplementation, especially after recovery from apnoea. Therefore, 3% O_2_ and 65% O_2_ are rather reflecting extremes at either end. Another important point to consider is the difference in immune responses due to gestational age, because very preterm infants below 29 weeks of gestation are those with the highest risk to develop BPD (11, 12, 14). Although, this is the first study analysing macrophage immune responses in a gestational age dependent manner, we enrolled a convenience sample that was limited to the gestational age group of 30-34 weeks due to limited sample amount and immune cell numbers in cord blood below 30 weeks of gestation. Earlier studies have shown that while neonatal monocytes and dendritic cells secrete abundant IL-23, levels fall drastically below 29 weeks of gestation (53). Similarly, levels of LPS-induced pro-inflammatory cytokines fall drastically in monocytes at a low gestational age (29). Therefore, a different immune response of macrophages below 30 weeks of gestation is a possibility, which might be a factor for the increased BPD risk. Another possibility would be that maturation of pro-inflammatory responses at 30-34 corrected gestational age could coincide with the development of BPD signs even in preterm infants born below 30 weeks of gestation. This hypothesis is supported by the post-natal maturation towards hyper-inflammatory TLR responses (54) and consistent with the model of myeloid differentiation during the third trimester of gestation (3). Future studies need to include postnatal peripheral blood samples preceding BPD in infants at risk in order to include the aspect of early developmental trajectories.

## Conclusion

Taken together, our findings indicate that there are gestational age-dependent differences in human MФ function, including the capacity for exaggerated inflammatory responses by preterm MФ following sequential exposure to stimuli that serve as risk factors for BPD. Our model serves as a useful tool for studying the sustained inflammatory response of infant lung disease *in vitro* with one of the key effector cell types involved in BPD. Moreover, we demonstrated an oxygen concentration-dependent downregulation of two major transcriptional regulators, Egr2 and Gfi1, in preterm MФ, suggesting that these may be involved in driving the pro-inflammatory MФ phenotype of preterm infants. Further studies are needed to verify the importance and mechanisms of Egr2 and Gfi1 regulation and to investigate whether the latter could also serve as therapeutic targets in BPD patients.

## Data Availability Statement

The datasets presented in this study can be found in online repositories. The names of the repository/repositories and accession number(s) can be found below: https://ega-archive.org, EGAS00001004974.

## Ethics Statement

The studies involving human participants were reviewed and approved by Local committee on research in human subjects at the University of Lübeck (IRON AZ 15-304). Written informed consent to participate in this study was provided by the participants’ legal guardian/next of kin.

## Author Contributions

NT, JP, and CH contributed to the conception and design of the study. CH, JP, and AHa contributed to recruitment of patients. NT performed cell culture experiments and graphical and statistical analysis. JW, MW, KG, WG, EH, and AHi contributed to graphical and statistical analysis and data interpretation. AK and HB contributed to design of RNA sequencing samples and performing the analysis of those samples. NT wrote the first draft of the manuscript. JP, CH, JW, AHa, AHi, KG, MW, WG, EH, AK, and HB edited the manuscript. All authors contributed to manuscript revision, read and approved the submitted version.

## Funding

NT was supported by a grant of the Friedrich-Ebert-Stiftung (financed by the German Ministry of Education and Research, BMBF). JP was supported by a grant of the German Centre of Infection Research (DZIF; financed by the German Ministry of Education and Research, BMBF). AH was supported by grants from the IRTG 1911 (financed by the German Society of Research, DFG). CH was supported by the DFG (IRoN study; HA-6409-5/1), the BMBF (PRIMAL clinical study) and the Lübeck-Hilfe-für krebskranke Kinder e.V. HB acknowledges funding by the Deutsche Forschungsgemeinschaft (DFG, German Research Foundation) under Germany`s Excellence Strategy – EXC 22167-390884018”. MW was supported by the German Center for Lung Research (DZL, German Ministry of Education and Research, BMBF).

## Conflict of Interest

The authors declare that the research was conducted in the absence of any commercial or financial relationships that could be construed as a potential conflict of interest.

## Publisher’s Note

All claims expressed in this article are solely those of the authors and do not necessarily represent those of their affiliated organizations, or those of the publisher, the editors and the reviewers. Any product that may be evaluated in this article, or claim that may be made by its manufacturer, is not guaranteed or endorsed by the publisher.
