# Supplementary material for: Hyperoxia/Hypoxia Exposure Primes a Sustained Pro-Inflammatory Profile of Preterm Infant Macrophages Upon LPS Stimulation
Source: Front Immunol. 2021 Nov 18;12:762789. doi: 10.3389/fimmu.2021.762789 (PMC8637891; doi:10.3389/fimmu.2021.762789)

Supplementary Material

Hyperoxia/hypoxia exposure primes a sustained pro-inflammatory profile of preterm infant macrophages upon LPS stimulation

Nele Twisselmann[1#], Julia Pagel[1,2], Axel Künstner[3], Markus Weckmann[4,5], Annika Hartz[1], Kirsten Glaser[6], Anne Hilgendorff[7], Wolfgang Göpel[1], Hauke Busch[3], Egbert Herting[1], Jason B. Weinberg[8,9], Christoph Härtel[10*]

[1]Department of Pediatrics Neonatology, University of Lübeck and University Medical Center Schleswig-Holstein, Lübeck, Germany;

[2]Department of Infectious Diseases and Microbiology, University of Lübeck and University Medical Center Schleswig-Holstein, Lübeck, Germany;

[3]Medical Systems Biology Group, Institute of Experimental Dermatology, University of Lübeck and University Medical Center Schleswig-Holstein, Lübeck, Germany;

[4]Department of Pediatrics Pneumology & Allergology, University Medical Center Schleswig-Holstein, Lübeck, Germany; Airway Research Center North (ARCN);

[5]Member of the German Center for Lung Research (DZL), Lübeck, Germany;

[6]Center for Pediatric Research, Division of Neonatology, Department of Women‘s and Children‘s Health, University of Leipzig Medical Centre, Leipzig, Germany;

[7]Center for Comprehensive Developmental Care (CDeCLMU), Member of the German Center for Lung Research (DZL), Hospital of the Ludwig-Maximilians University (LMU), CPC-M bioArchive, Munich, Germany;

[8]Department of Pediatrics, University of Michigan, Ann Arbor, Michigan, United States;

[9]Department of Microbiology and Immunology, University of Michigan, Ann Arbor, Michigan, United States;

[10]Department of Pediatrics, University of Würzburg, Würzburg, Germany

[#] Heinrich-Pette-Institute, Leibniz-Institute for Experimental Virology, Hamburg, Germany

*** Correspondence:**
Department of Pediatrics University of Würzburg
Josef-Schneider-Strasse 2
D-97080 Würzburg
Ph:+49-93120127831
e-mail: haertel_c1@ukw.de

Keywords: preterm infants, sustained inflammation, macrophages, hyperoxia, hypoxia, infection, bronchopulmonary dysplasia

Supplementary materials and methods

Supplementary Method 1: Morphology

To compare the morphology of monocyte-derived macrophages (MΦ) from preterm and term infants as well as adults after differentiation, three phase contrast pictures per experiment and donor were obtained using a fluorescence microscope at 20x magnification.

Supplementary Method 2: Intracellular flow cytometry staining after differentiation

The staining of surface proteins was performed as described in the method section for flow cytometry. Directly after differentiation, $5x10^4$ to $1x10^5$ MΦ were transferred into a FACS tube (first panel). When staining intracellular proteins, $2x10^5$ to $4x10^5$ cells were transferred per FACS tube (second panel). Anti-human antibodies against surface proteins were added; for the first panel: 1:33 PE conjugated CD14 (Biolegend, San Diego, CA, USA), 1:33 APC conjugated CD45 (Biolegend, San Diego, CA, USA), and 1:20 pre-diluted Fixable Viability Dye; for the second panel: 1:25 APC conjugated CD11b (Biolegend, San Diego, CA, USA), and 1:20 pre-diluted Fixable Viability Dye. For the first panel, cells were resuspended in 150 µL FACS buffer for flow cytometric analysis after the last washing step. For the second panel, intracellular staining was performed using the Foxp3/Transcription Factor Staining Buffer Set (eBioscience, Thermo Fisher Scientific, Waltham, MA, USA) according to the manufacturer's instructions, except for the following: working solution was diluted 1:8 and incubated 30 min in the dark. To block unspecific binding sites intracellularly, 20 µL FcR blocking reagent were added to all tubes after a washing step, samples were pulse vortexed and incubated for 15 min at room temperature in the dark. Next, an anti-human FITC conjugated CD68 antibody (Biolegend, San Diego, CA, USA) was added in a 1:25 dilution, samples were pulse vortexed and incubated for another 30 min at room temperature in the dark. Washing samples was repeated by adding 2 mL 1x permeabilization buffer, centrifugation at 1500 rpm for 5 min at room temperature and discarding supernatant by inverting the tube in one continuous motion. As a last step, 150 µL flow cytometry staining buffer was added to each sample.

Supplementary Method 3: Indirect immunofluorescence

To assess protein expression of transcription factors and their translocation to the nucleus, immunostaining was used. Chamber slides were coated with 20-30 µg/mL Cell-Tak (Corning Incorporated, Corning, New York, USA) in 100 µL sodium bicarbonate buffer for 20 min at room temperature. Wells were then washed twice with 500 µL sterile distilled water and air-dried for 1 h under sterile conditions. After differentiation, $5x10^4$ MΦ per mL were seeded in 400 µL stimulation medium per coated well. After a 1 to 2 h resting and adherence phase in an incubator containing an atmosphere of 5% CO_2 and 21% O_2 at a temperature of 37 °C, chamber slides were transferred to different incubators with 5% CO_2 at 37 °C containing the desired O_2 conditions, including 21% atmospheric O_2, 3% O_2 (incubator with N2-regulation) and 65% O_2 (air-tight sealed chamber filled with gas mixture), and incubated for 5 h. In the following description, volumes were specified per chamber. Immediately after the 5 h incubation period, the supernatant was discarded, and cells were fixed by adding 200 µL fixing buffer (3% PFA in 2/3 destilled water and 1/3 PBS buffer) for 30 min at room temperature. Afterwards, cells were washed three times with 200 µL washing solution (10mM glycine in PBS) and stored in PBS buffer at 4 °C overnight. On the next day, 200 µL permeabilization buffer (0.1% Triton-X 100 in PBS buffer) were added and cells were incubated 5 min at room temperature. Following permeabilization and three washes with 200 µL PBS buffer per wash, non-

specific binding sites on cells were blocked for 30 min at room temperature using 200 µL immunostaining buffer (1% BSA, 0.01% NaN_3 1% human serum in PBS buffer). Afterwards, immunostaining buffer was completely removed, and cells were incubated with 100 µL primary anti-human antibody diluted 1:50 (HIF-1α, rabbit, Abcam, Cambridge, UK) or 1:100 (Nrf2, rabbit, Abcam, Cambridge, UK) in immunostaining buffer for 1 h at room temperature. Next, cells were washed three times with 200 µL PBS buffer and then incubated with 200 µL 1:1000 diluted secondary anti-rabbit antibody (IgG, goat 594nm, Cell Signaling Technology, Danvers, MA, USA) for 30 min at 37 °C in the dark. After repeating the washing step three times using 200 µL PBS buffer per wash, cells were incubated with 200 µL DAPI solution (1 ng/mL) for 15 min at room temperature in the dark and washed two times with 200 µL PBS buffer per wash. Following the staining procedure, PBS buffer and chambers were removed to let the slides dry. Using one drop of mounting solution, slides were covered with a coverslip and sealed using nail polish. Slides were stored at 4 °C for up to 2 days to allow them to dry. Three to four microscopy pictures were obtained per condition at 100x magnification using oil on the objective of the fluorescence microscope. To obtain comparable pictures, the same exposure time was used for each image. Afterwards, pixel intensity of the red channel was analyzed in the nucleus of Nrf2- or HIF-1α-stained cells using ImageJ. Results represent 3 to 4 microscopy pictures per O_2 condition for each experiment, including 8 to 12 cells analyzed per picture.

Supplementary Figures

Supplementary Figure 1: Characteristics of monocyte-derived macrophages (MΦ) from preterm and term infants as well as adults after differentiation using macrophage colony-stimulating factor (M-CSF). After differentiation for 6 days using M-CSF, A) viability was assessed by flow cytometry (n=6, median with range), B) morphology was compared using microscopy images (scale bar 50 µm, one representative picture per group from n=3 independent experiments of three different donors), and C) marker expression frequencies of CD14, CD68 and CD11b were determined using flow cytometry (D) including a representative histogram of the preterm, term and adult group (n=3 independent experiments of three different donors, median with range).

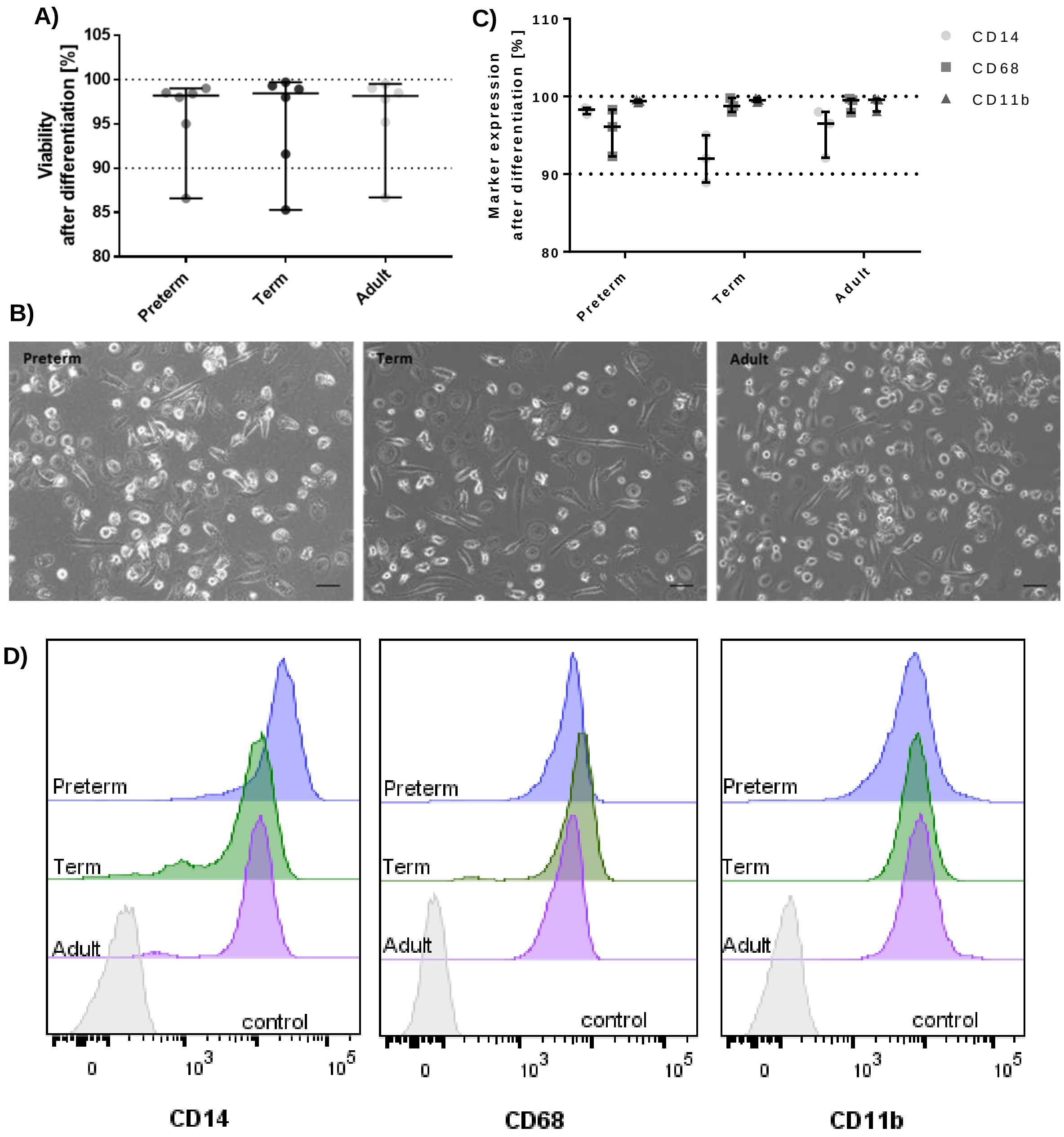

Supplementary Figure 2: Viability and expression of polarization surface marker on preterm, term, and adult macrophages (MΦ) after LPS stimulation. LPS-stimulated MΦ of preterm infants compared to term and adult MΦ after incubation for 72 h (48 h 21% O_2 followed by 24 h at 21% O_2 with or without LPS). A) Viability of MΦ depicted in percentage of all single cells (%; n=4 independent experiments with cells of four different donors (same as Figure 3), mean ± SEM). B-C) Surface expression of B) CD80, C) CD206, and D) CD200R depicted in mean fluorescence intensity [MFI] of surface proteins on macrophages in all three groups assessed by flow cytometry. (n=4 independent experiments with cells of four different donors (same as Figure 3), box plots: median ± 25th to 75th percentiles and minimum to maximum, ANOVA followed by Holm-Sidak's test, *p<0.05, **p<0.01, and ***p<0.001).

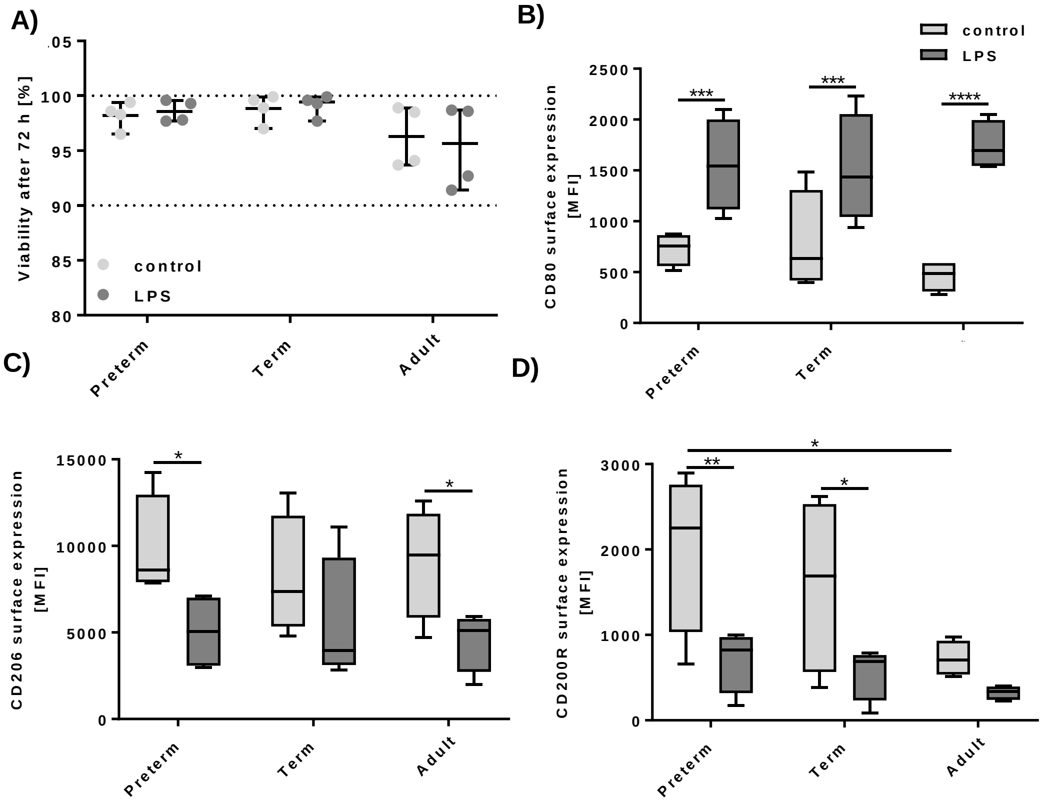

Supplementary Figure 3: 62 differentially regulated transcription factors of LPS-stimulated preterm macrophages (MΦ) compared to term MΦ. Preterm and term MΦ were incubated for 72 h (48 h 21% O_2 followed by 24 h 21% O_2 with or without LPS)**.** The bar graph combines the effect of gestational age and stimulatory condition by comparing the difference of LPS stimulation with control (21% O_2) from preterm MΦ to the same difference from term MΦ. The TRAP analysis used input of differentially regulated genes (unpaired t-test, $p_{adj} < 0.05$) form LPS-stimulated and unstimulated preterm and term MΦ. Upper transcription factors lead to downregulated pathways depicted in Figure 5D, whereas lower transcription factors lead to upregulated pathways depicted in Figure 5C (regulator cutoff: $p_{adj}<0.01$). 50 of 62 transcription factors were further regulated comparing the difference of double-hit conditions with LPS alone from preterm MΦ to the same difference from term MΦ (25 factors depicted in green for both 65% and 3% double-hits; another 25 factors depicted in blue only for 3% double-hit; n=4 independent experiments with cells of four different donors (same as Figure 5)).

Preterm MΦ (LPS compared to control)
minus term MΦ (LPS compared to control)

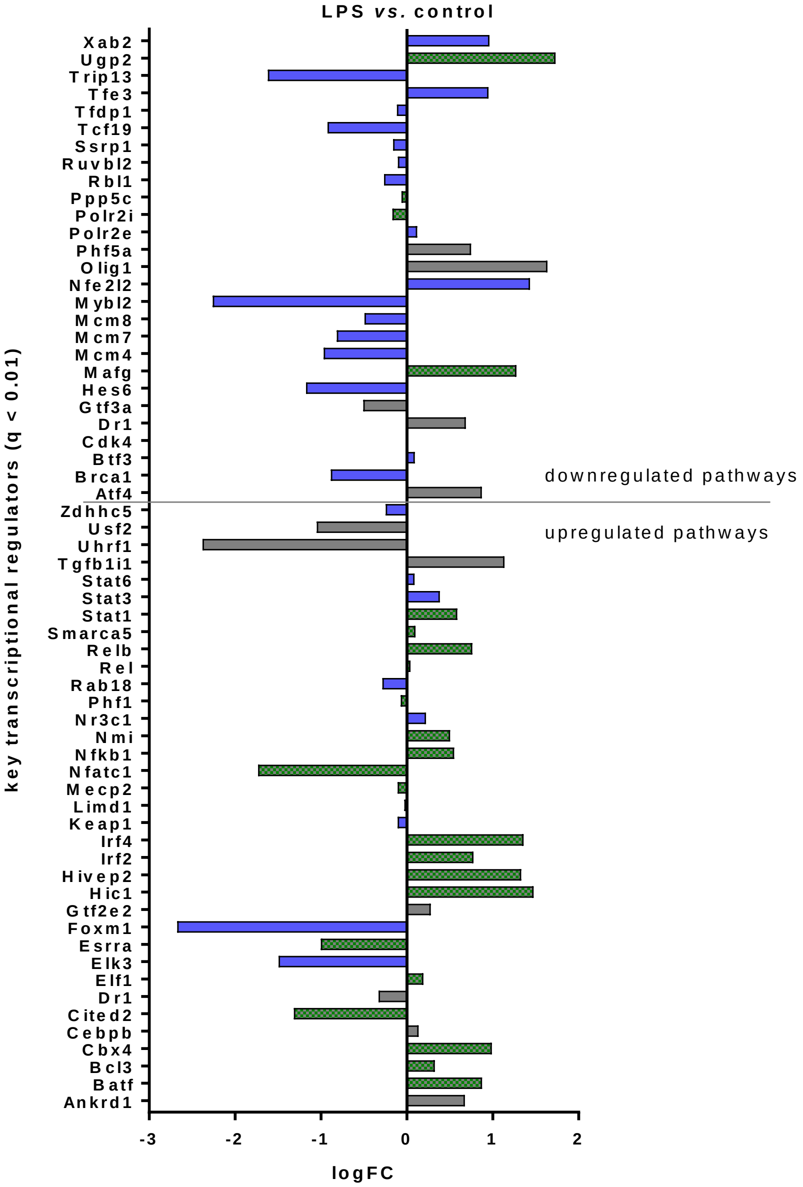

Immune response of preterm infant macrophages

Supplementary Figure 4: Oxygen sensing of macrophages (MΦ). Neonatal MΦ were incubated for 5 h at various oxygen conditions, namely: 21% O_2 as control, 65% O_2 (hyperoxia), and 3% O_2 (hypoxia). MΦ were stained for fluorescence microscopy using antibodies against Nuclear factor-like (Nrf)2 protein and Hypoxia inducible factor (HIF)-1α protein. A and C) Pixel intensity in the nucleus (blue) of Nrf2 (red) stained cells. B and D) Pixel intensity in the nucleus (blue) of HIF-1α (red) stained cells. Box plots represent 3 to 4 microscopy pictures per O_2 condition for each experiment, including 8 to 12 cells analyzed per picture (scale bar 10 µm, one representative picture per group from n=3 independent experiments using different donors, box plots: median ± 25th to 75th percentiles and minimum to maximum, ANOVA followed by Holm-Sidak's test, *p<0.05, **p<0.01).

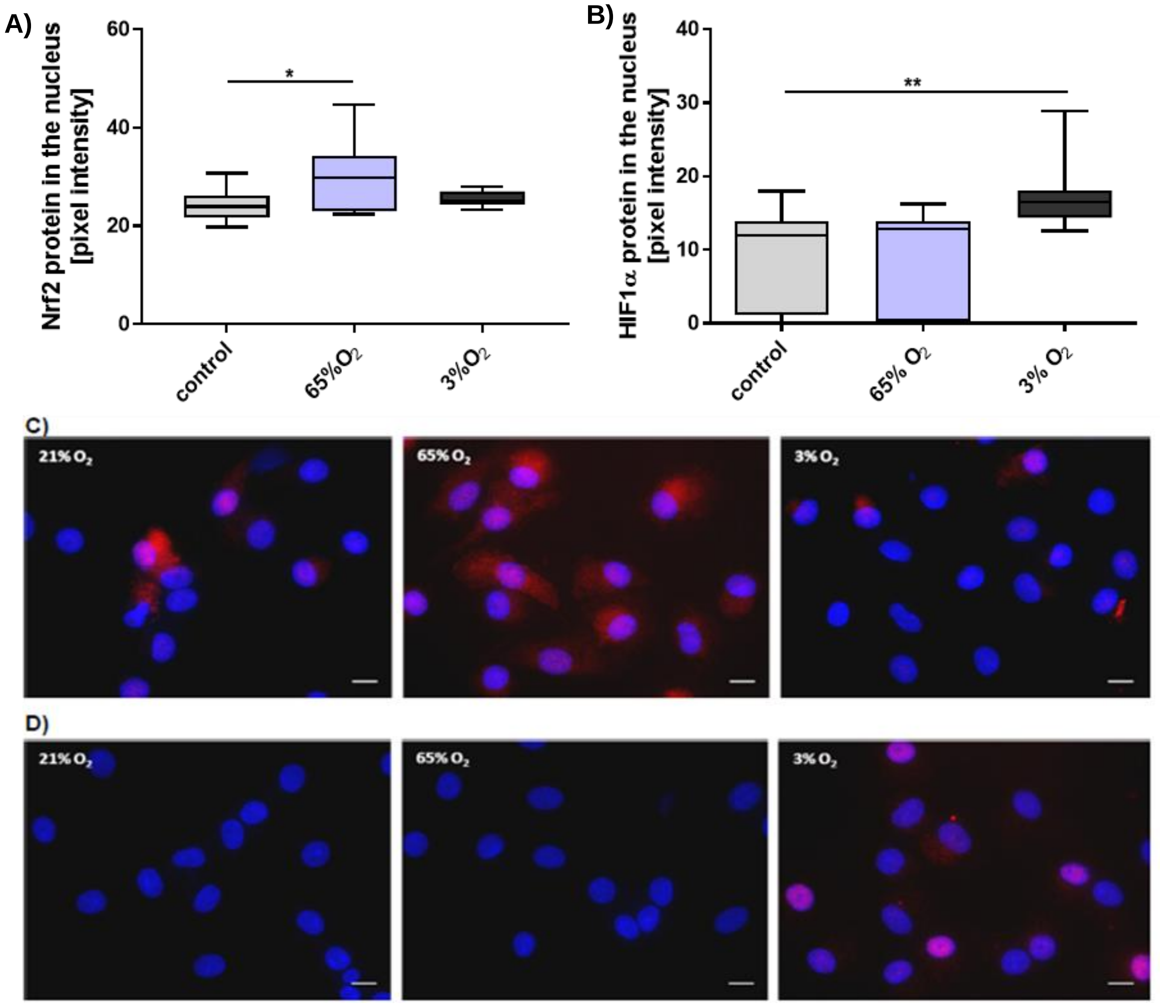

Supplementary Figure 5: Cytokine release from preterm, term and adult macrophages (MΦ) primed with various O_2 concentrations followed by LPS. IL-23 and IL-10 release after O_2 exposure only compared to control (21% O_2) (A and B) and after priming with 65% O_2 or 3% O_2 followed by LPS compared to LPS alone (C and D) incubated for 72 h (48 h 65% O_2 or 3% O_2 followed by 24 h 21% O_2 with or without LPS). Cytokine release was measured in the supernatant of all three groups using a cytokine bead array (n=5 independent experiments with supernatants of five different donors (same as Figure 6; control and LPS values were re-used from Figure 2 because oxygen and double-hit conditions were included in those experiments), box plots: median ± 25th to 75th percentiles and minimum to maximum, ANOVA followed by Holm-Sidak's test, *p<0.05, **p<0.01, ***p<0.001, and ****p<0.0001).

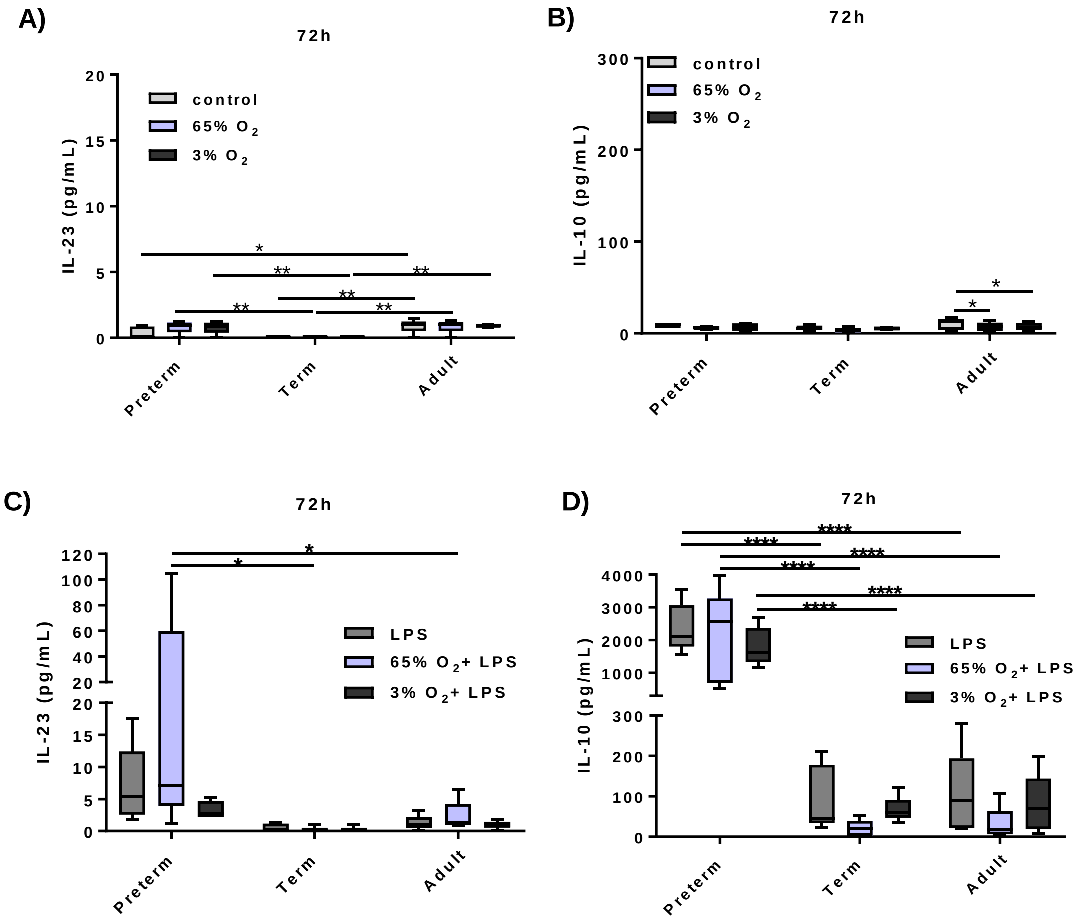

Supplement: Supplementary file 1 [file DataSheet_1.pdf]
